# Systematic review and meta-analysis: multimodal functional and anatomical neural alterations in autism spectrum disorder

**DOI:** 10.1186/s13229-024-00593-6

**Published:** 2024-04-04

**Authors:** Zixuan Guo, Xinyue Tang, Shu Xiao, Hong Yan, Shilin Sun, Zibin Yang, Li Huang, Zhuoming Chen, Ying Wang

**Affiliations:** 1 Medical Imaging Center, The First Affiliated Hospital of Jinan University, Guangzhou, China; 2 Department of Rehabilitation Medicine, The First Affiliated Hospital of Jinan University, Guangzhou, Guangdong China

**Keywords:** Meta-analysis, Autism spectrum disorder, Resting-state functional imaging, Voxel-based morphometry, Gray matter volume

## Abstract

**Background:**

This meta-analysis aimed to explore the most robust findings across numerous existing resting-state functional imaging and voxel-based morphometry (VBM) studies on the functional and structural brain alterations in individuals with autism spectrum disorder (ASD).

**Methods:**

A whole-brain voxel-wise meta-analysis was conducted to compare the differences in the intrinsic functional activity and gray matter volume (GMV) between individuals with ASD and typically developing individuals (TDs) using Seed-based *d* Mapping software.

**Results:**

A total of 23 functional imaging studies (786 ASD, 710 TDs) and 52 VBM studies (1728 ASD, 1747 TDs) were included. Compared with TDs, individuals with ASD displayed resting-state functional decreases in the left insula (extending to left superior temporal gyrus [STG]), bilateral anterior cingulate cortex/medial prefrontal cortex (ACC/mPFC), left angular gyrus and right inferior temporal gyrus, as well as increases in the right supplementary motor area and precuneus. For VBM meta-analysis, individuals with ASD displayed decreased GMV in the ACC/mPFC and left cerebellum, and increased GMV in the left middle temporal gyrus (extending to the left insula and STG), bilateral olfactory cortex, and right precentral gyrus. Further, individuals with ASD displayed decreased resting-state functional activity and increased GMV in the left insula after overlapping the functional and structural differences.

**Conclusions:**

The present multimodal meta-analysis demonstrated that ASD exhibited similar alterations in both function and structure of the insula and ACC/mPFC, and functional or structural alterations in the default mode network (DMN), primary motor and sensory regions. These findings contribute to further understanding of the pathophysiology of ASD.

**Supplementary Information:**

The online version contains supplementary material available at 10.1186/s13229-024-00593-6.

## Introduction

Autism spectrum disorder (ASD) is a neurodevelopmental disorder characterized by deficits in social interaction and communication, repetitive and restricted patterns of behavior, and interests and activities [1]; it is more common in males [1]. Individuals with ASD in the absence of intellectual disability (full-scale intelligence quotient [IQ] > 80) tend to have better emotional and cognitive functioning [2]. They have been reported to have milder autism symptomatology and comparable ability to typically developing individuals (TDs) [2]. The increasing prevalence of ASD, estimated at 1 in 44 eight-year-old children, is an acknowledged public health crisis, posing a huge social and economic burden on society [3–5]. Unfortunately, early detection is often abstruse due to the heterogeneous symptomology and gradual course [1, 5], and the underlying neuropathology remains unknown [4, 6].

Accumulating evidence reveals that functional and structural brain abnormalities have been considered as a promising mechanism for the pathophysiology of ASD over the past decade [4, 6]. With continuing efforts toward data-sharing and classification analyses within the field of ASD research, meta-analyses can be useful and unbiased methodological approaches that will provide convincing information [7]. Numerous existing task-based functional meta-analyses have reported ASD-associated hyper- and/or hypoactivation in the anterior cingulate cortex/medial prefrontal cortex (ACC/mPFC), insula, and frontotemporal regions during nonsocial and social cognitive processing [7–11]. Intriguingly, a limited number of meta-analyses have been conducted based on resting-state functional imaging, a more effective and stable method to investigate intrinsic and task-free brain activity at baseline [12]. The increased neural activity results in two primary outcomes: alterations in oxygen concentration (referred to as blood-oxygen-level-dependent [BOLD] [13] signal) and regional cerebral blood flow (CBF) [14]. BOLD signals, can be measured using functional magnetic resonance imaging (fMRI), consist of three local metrics: amplitude of low-frequency fluctuation (ALFF), fractional ALFF (fALFF), and regional homogeneity (ReHo). ALFF/fALFF reflects the intensity of BOLD signals oscillations within a specific low-frequency range (0.01–0.08 Hz) at the single-voxel level [15]. ReHo represents the time-series similarity of BOLD signals between a given voxel with its neighboring voxels [16]. CBF can be quantified using endogenous arterial water (arterial spin labeling [ASL] [17]) or exogenous radioisotopes through imaging techniques such as fMRI, single-photon emission computed tomography (SPECT), and positron emission tomography (PET). All these functional modalities undoubtedly reflect intrinsic functional brain activity at baseline, and the combination contributes towards a more comprehensive assessment of brain dysfunction. Previous meta-analyses combined the aforementioned functional modalities and provided a comprehensive assessment of disorder-related effects on psychiatric disorders, including ASD [6], major depressive disorder (MDD) [18, 19], bipolar disorder (BD) [12], substance use disorder [20], and anorexia nervosa [21]. Only one ASD-related resting-state functional meta-analysis has been conducted to date, suggesting functional aberrations in language network, default mode network (DMN), and cerebellum; however, the study failed to conduct subgroup analyses due to relatively limited included studies [6]. Hence, it was necessary to update the meta-analyses to expand and/or modify the findings of previous studies [12, 21].

Additionally, voxel-based morphometry (VBM) and surface-based cortical morphometry (SBM) are common automatic analytical methods in neuroanatomy. VBM is the most widely used whole-brain method to measures gray matter volume (GMV) unbiasedly [12, 22]. Although numerous VBM studies focused on the structural brain differences in ASD, the results are inconsistent and poorly replicated due to heterogeneous clinical samples, protocol variability, medical comorbidity and flexible analyses [5, 23], which have made a firm neural characterization challenging. To date, extant VBM-based meta-analyses have reported GMV alterations in ASD, predominantly in the insula, ACC/mPFC, cerebellum and frontotemporal regions [11, 24–28]. Moreover, the Enhancing Neuro Imaging Genetics through Meta-Analysis (ENIGMA) consortium also reported smaller subcortical volumes and abnormal cortical thickness in the frontotemporal regions in ASD [29–31]. However, the ENIGMA uses the FreeSurfer software, which segments brain regions based on probabilistic information from a predefined atlas compared to VBM’s voxel-wise registration [32]. The differences in these methodological approaches may lead to diverging results [32]. To date, no multimodal meta-analysis has been conducted to date to detect task-free functional activity changes in combination with the structural alterations in individuals with ASD, which may elucidate consensus functional and structural regional aberrations and provide more information on the neuropathological mechanisms in ASD.

Therefore, we performed a multimodal and updated whole-brain voxel-wise meta-analysis to explore the most robust findings across numerous existing resting-state functional imaging studies and VBM studies using the Seed-based *d* Mapping (SDM) software. Specifically, we separately performed resting-state voxel-based physiology and VBM meta-analysis, as well as subsequent overlapping of functional and structural abnormalities. Additionally, the subgroup (e.g., male sex, age, and ASD without any psychiatric comorbidities) meta-analyses were conducted to check the robustness and heterogeneity of the main results, and meta-regression analyses were performed to assess the potential associations with clinical variables. We hypothesized based on existing studies that the functional activity and structural abnormalities were located in the frontotemporal regions, ACC/mPFC, and insula.

## Method

### Study selection and quality assessment

The present meta-analysis was conducted in accordance with the Preferred Reporting Items for Systematic reviews and Meta-Analyses (PRISMA) guidelines (registration number is: CRD42023379003) [33]. A systematic and comprehensive search of studies published between January 1990 and September 2022 was carried out on Embase, PubMed, Web of Science, WanFang, Chinese National Knowledge Infrastructure, and Vip databases. We used the following keywords to search for resting-state fMRI studies and VBM studies, respectively: (1) (“autism” OR “autism spectrum disorder” OR “ASD” OR “asperge” OR “asperger syndrome” OR “communication disorder” OR “autistic” OR “autistic disorder”) AND (“amplitude of low-frequency fluctuation” OR “ALFF OR low-frequency fluctuation” OR “LFF” OR “amplitude of low-frequency oscillation” OR “LFO” OR “fractional amplitude of low-frequency fluctuation” OR “fALFF” OR “reho” OR “regional homogeneity” OR “ReHo” OR “cerebral blood flow” OR “CBF” OR “positron emission tomography” OR “PET” OR “single photon emission computed tomography” OR “SPECT” OR “arterial spin labeling” OR “ASL”); (2) (“autism” OR “autism spectrum disorder” OR “ASD” OR “asperge” OR “asperger syndrome” OR “communication disorder” OR “autistic” OR “autistic disorder”) AND (“structural magnetic resonance imaging” OR “morphometry” OR “voxel-based” OR “voxel-wise” OR “voxel-based morphometry” OR “VBM” OR “high-resolution imaging” OR “structural neuroimaging” OR “grey matter” OR “gray matter”). Furthermore, an additional check within the reference list of included studies and previous ASD-related meta-analyses was performed to identify other relevant studies.

**Fig. 1:**
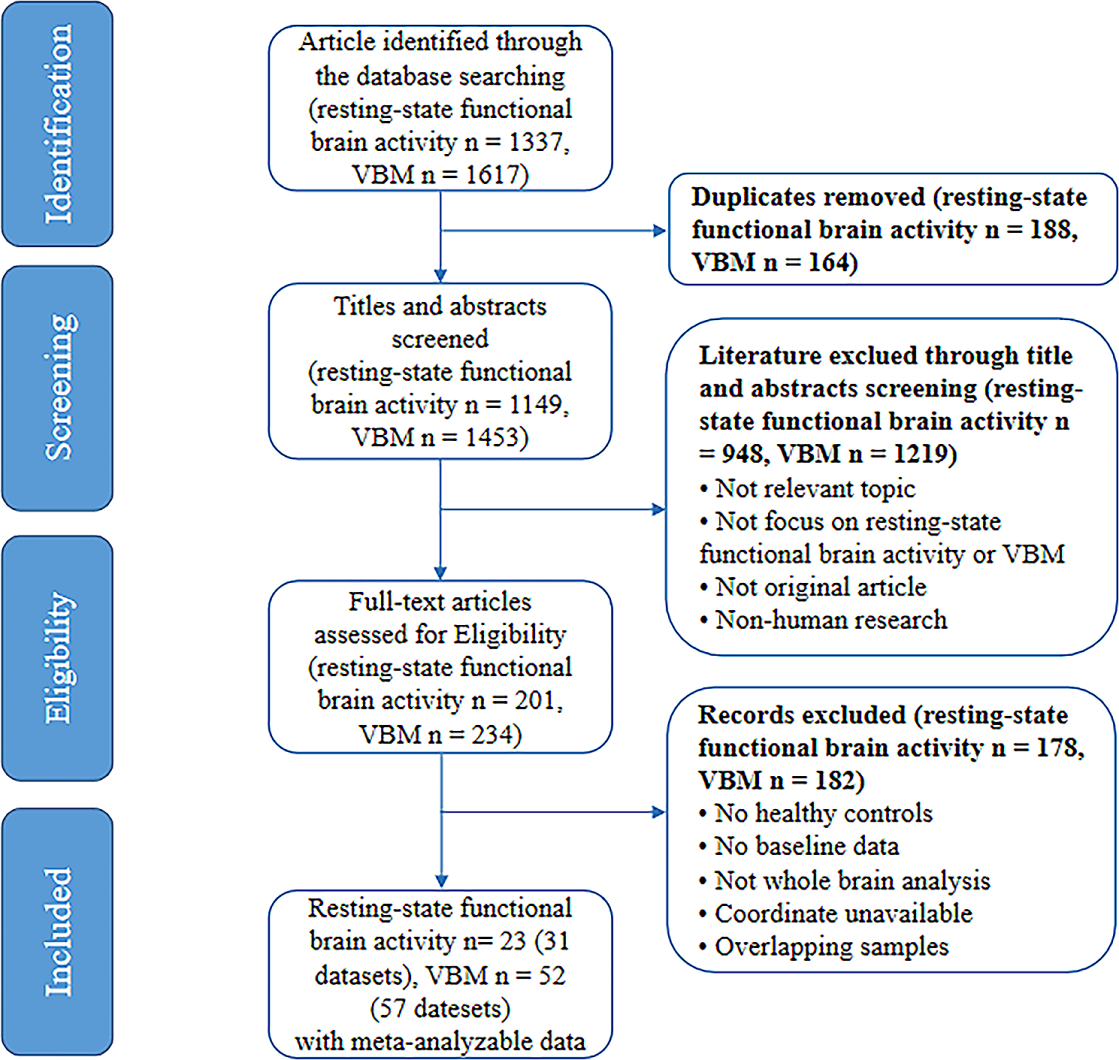
PRISMA flowchart outlining the article screening and selection process. ***Note***: PRISMA = Preferred Reporting Items for Systematic reviews and Meta-Analyses; ASD = autism spectrum disorder; VBM = voxel-based morphometry

Studies had to fulfill the following inclusion criteria: (1) conducted functional and/or structural neuroimaging analysis comparing ASD with TD at the whole-brain level; (2) original research published in English or Chinese journals; (3) three-dimensional standard stereotactic coordinates (Montreal Neurological Institute (MNI) or Talairach) were reported. Studies were excluded if (1) the study sample overlapped with those of another publication (in which case the publication with the largest sample size was preferentially selected). The publications from open databases were excluded in case sample duplication was contained; (2) individuals with ASD with comorbid neurological diseases; (3) analysis based on the region of interest (ROI). A flowchart for the inclusion and exclusion of studies was shown in Fig. 1.

Consistent with the previous meta-analyses [6, 21], the quality of each included study was assessed using a 10-point checklist (Table S1), which focused on the clinical and demographic aspects of the study samples and the imaging methodology. The checklist was divided into three parts: participants (items 1–4), imaging methodology and analysis (items 5–8), and results and conclusions (items 9 and 10). Two authors (Z.X.G. and X.Y.T.) conducted the literature search, study selection, and quality assessment independently, and the third investigator (Y.W.) would make the final decision if the three has any disagreements.

### Main meta-analysis of functional differences

Meta-analysis of functional (i.e., ALFF, fALFF, ReHo, CBF) group differences was conducted using the SDM software (www.sdmproject.com). Briefly, the SDM, a voxel-based meta-analysis software, used reported peak coordinates and their statistics (e.g., t-values) to recreate an effect-size signed map of the difference between individuals with ASD and TDs for each original study [6, 34]. We selected the “Functional MRI or PET” modality and the “gray matter” correlation template within the “gray matter” mask to improve the accuracy of effect size maps. Next, the maps were then consolidated in a standard random-effects model weighing sample size (i.e., studies with larger sample size or lower variability contribute more), intra-study variability, and between-study heterogeneity, and multiple imputations are pooled according to Rubin’s rules [35]. According to the recommendations of the developer of SDM and previous studies, an uncorrected *p* < 0.005 threshold with minimum cluster extent > 50 voxels and SDM-Z > 1 was used to reduce the possibility of false positive results and to optimally balance false positives and negatives [6, 34]. In addition, MRIcron software package (www.mricro.com/mricron/) was used to visualize SDM maps.

### Main meta-analysis of structural differences

Consistent with the above analysis, a meta-analysis of structural (GMV) group differences were also performed in SDM software with the similar procedure steps (Supplementary material 1).

### Overlapping of the functional and structural differences

Areas of overlapping functional and structural abnormalities were assessed by conjunction analysis through the multimodal meta-analysis in SDM software. This analysis is conceptually the same as conducting the simple overlap of the meta-analytical maps from individual meta-analyses (i.e., to find the regions presenting differences both at the structural and functional level), but it takes into account the error in the *p*-values [36, 37].

### Subgroup meta-analysis

To investigate underlying clinical and methodological heterogeneity, we redid the meta-analyses in the following subgroups: (1) male; (2) childhood (0–12 years old); (3) adolescence (13–17 years old); (4) adult ASD (age > 18 years old); (5) ASD without any psychiatric comorbidities; (6) excluding the insufficient sample size of individuals with ASD (*n* < 20) or uncorrected for results; (7) imaging techniques. Subgroup meta-analysis was not performed when the number of datasets was insufficient (*n* < 10), as suggested for SDM meta-analyses [10, 12, 38].

### Analyses of jackknife sensitivity, heterogeneity and publication bias

To evaluate the repeatability of results, a whole-brain voxel-based jackknife sensitivity analysis was performed by iteratively repeating the same analyses, discarding one dataset each time. If a brain region is still considerable in all or most of the study combinations, the finding is considered highly repeatable.

Residual heterogeneity (*I*^2^ statistic) of included studies was performed to evaluate robustness of results, with *I*^2^ < 50% considering low heterogeneity [39]. As for potential publication bias, funnel plots were created for visual inspection and Egger’s tests were performed [40]. An asymmetric plot and *p*-value < 0.05 were considered statistically significant for publication bias.

### Meta-regression analyses

A meta-regression analyses was conducted to assess potential effects of clinicalvariables (e.g., age, percentage of males, Autism Diagnostic Interview—Revised [ADI-R] score [41], IQ) on the functional and structural results. According to previous studies, [42] a more conservative threshold of *P* < 0.0005 was selected in order to minimize the reporting of spurious findings. And we discarded findings in regions other than those detected in the main meta-analyses. Results are reported in MNI space.

## Results

### Studies included in the meta-analysis

A flowchart for the process of systematic literature search and assessment of eligible studies is shown in Fig. 1. We finally included 23 independent studies with 31 datasets (6 datasets for ALFF, 2 datasets for fALFF, 16 datasets for ReHo, and 7 datasets for CBF) for resting-state functional imaging, comprising 786 individuals with ASD and 710 TDs, and 52 independent studies (57 datasets) for VBM, comprising 1728 individuals with ASD and 1747 TDs (Table 1). In statistics, we excluded subjects without complete information on age, sex, IQ or ADI-R. Tables S2 and S3 briefly summarize the demographic data, quality score, and clinical and imaging characteristics of the studies.

**Table 1:** Demographic information of meta-analysis samples

	ASD	TD	*p* value
**Resting-state functional imaging**			
Sample, n	786	710	0.540
Mean age, y	11.07 ± 7.28	11.33 ± 6.96	0.869
Male/Female	680/106	580/130	0.011^a^
Mean FSIQ	93.42 ± 17.02	107.31 ± 11.09	0.028^a^
Mean ADI-R	41.55 ± 5.32	NA	NA
**VBM**			
Sample, n	1728	1747	0.630
Mean age, y	18.73 ± 9.33	19.13 ± 9.56	0.823
Male/Female	1516/212	1503/244	0.138
Mean FSIQ	99.76 ± 16.02	111.45 ± 6.51	< 0.001^a^
Mean ADI-R	39.48 ± 5.31	NA	NA

***Note***: ASD = autism spectrum disorder; TD = typically developing; FSIQ = full-scale intelligence quotient; ADI-R = Autism Diagnostic Interview—Revised; VBM = voxel-based morphometry

^a^Statistical significance

### Main functional meta-analysis

**Fig. 2:**
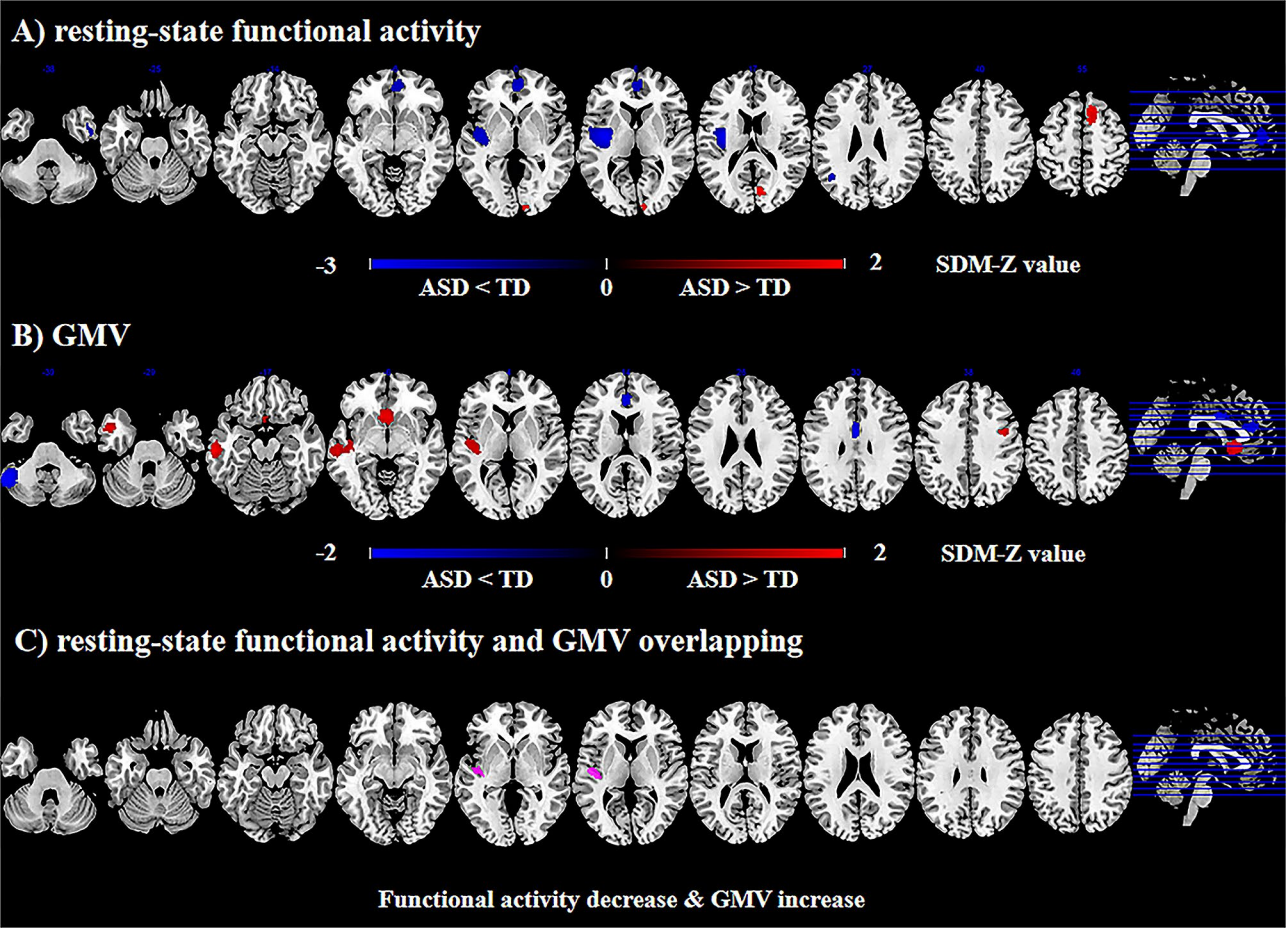
Meta-analyses results of difference between ASD and TD. ***Note***: (**A**) resting-state functional difference between ASD and TD; (**B**) GMV differences between ASD and TD; (**C**) Overlapping of resting-state functional activity differences and GMV differences. Areas with increased resting-state functional activity value or GMV value are displayed in red, and areas with decreased resting-state functional activity value or GMV value are displayed in blue. Areas with increased resting-state functional activity value and decreased GMV are displayed in purple. The color bar indicates the maximum and minimum SDM-Z values. ASD = autism spectrum disorder; TD = typically developing; SDM = seed-based *d* mapping; VBM = voxel-based morphometry; GMV = gray matter volume

The functional differences in individuals with ASD relative to TDs are shown in Fig. 2A; Table 2. Individuals with ASD displayed decreased resting-state functional activity in the left insula (extending to the left superior temporal gyrus [STG]), bilateral ACC/mPFC, left angular gyrus, and right inferior temporal gyrus (ITG), and increased resting-state functional activity in the right supplementary motor area (SMA) and precuneus.

**Table 2:** Meta-analyses results regarding resting-state functional and GMV difference between ASD and TD

Local Maximum					Cluster		Jackknife sensitivity	*I* ^2^	Egger’s test (*p* value)
Region	Peak MNI coordinate (x, y, z)	SDM-Z value	*p* value		No. of voxels	Breakdown (No. of voxels)			
**Functional difference**									
**ASD < TD**									
Left insula, BA 48	-40, -10, 10	-2.147	< 0.001		1156	Left insula, BA 48 (435) Left rolandic operculum, BA 48 (255) Left heschl gyrus, BA 48 (128) Left superior temporal gyrus, BA 48 (88)	31/31	No (*I*^2^ < 50%)	0.190
Right superior frontal gyrus, medial, BA 10	4, 50 ,0	-1.738	< 0.001		385	Right superior frontal gyrus, medial, BA 10, 11 (225) Left anterior cingulate / paracingulate gyri, BA 10, 32 (78) Left superior frontal gyrus, medial, BA 10 (60) Right anterior cingulate / paracingulate gyri, BA 10, 32 (17)	29/31	No (*I*^2^ < 50%)	0.857
Left angular gyrus, BA 39	-44, -56, 28	-1.712	< 0.001		76	Left angular gyrus, BA 39 (48)	29/31	No (*I*^2^ < 50%)	0.131
Right inferior temporal gyrus, BA 20	50, -2, -36	-1.471	0.003		63	Right inferior temporal gyrus, BA 20 (48) Right temporal pole, middle temporal gyrus, BA 20, 21 (15)	25/31	No (*I*^2^ < 50%)	0.075
**ASD > TD**									
Right supplementary motor area, BA 6, 11	8, 8, 52	1.138	0.002		239	Right supplementary motor area, BA 6, 8 (95)	30/31	No (*I*^2^ < 50%)	0.295
Right precuneus, BA 17, 18	10, -94, 4	1.150	0.002		73	Right precuneus, BA 17, 18 (59)	30/31	No (*I*^2^ < 50%)	0.949
**GMV difference**									
**ASD < TD**									
Left cerebellum, crus I	0, 40, 14	-1.383	< 0.001		653	Left cerebellum, crus I (287) Left cerebellum, crus II (270) Left cerebellum, hemispheric lobule VIIB (76)	56/57	No (*I*^2^ < 50%)	0.914
Left anterior cingulate / paracingulate gyri, BA 32	0, 40, 14	-1.204	0.001		187	Left anterior cingulate / paracingulate gyri, BA 24, 32 (141) Left superior frontal gyrus, medial, BA 32 (8)	53/57	No (*I*^2^ < 50%)	0.804
Left anterior cingulate / paracingulate gyri	0, 0, 30	-1.338	< 0.001		97	Left anterior cingulate / paracingulate gyri (32) Left median cingulate / paracingulate gyri 24, 23 (24) Right median cingulate / paracingulate gyri 23, 24 (17)	54/57	No (*I*^2^ < 50%)	0.051
**ASD > TD**									
Left middle temporal gyrus, BA 21	-60, -20, -14	1.807	< 0.001		789	Left middle temporal gyrus, BA 21, 20, 22, 48 (402) Left insula, BA 48 (64) Left superior temporal gyrus, BA 48 (61)	56/57	No (*I*^2^ < 50%)	0.095
Left olfactory cortex	-2, 20, -8	1.671	< 0.001		412	Left olfactory cortex, BA 25 (107) Right olfactory cortex, BA 25 (60)	56/57	No (*I*^2^ < 50%)	0.425
Left middle temporal gyrus, BA 20	-46, 4, -26	1.640	< 0.001		181	Left middle temporal gyrus, BA 21 (83) Left middle temporal gyrus, BA 20 (32)	56/57	No (*I*^2^ < 50%)	0.439
Right precentral gyrus	42, 2, 34	1.541	< 0.001		94	Right precentral gyrus, BA 6 (35)	54/57	No (*I*^2^ < 50%)	0.519

***Note***: GMV = gray matter volume; ASD = autism spectrum disorder; TD = typically developing; MNI = Montreal Neurological Institute; SDM = seed-based *d* mapping; BA = Brodmann area

### Main VBM meta-analysis

Compared with TDs, individuals with ASD displayed decreased GMV in the ACC/mPFC (extending to the bilateral median cingulate cortex [MCC]), and left cerebellum, and increased GMV in the left middle temporal gyrus (MTG) (extending to the insula and left STG), bilateral olfactory cortex, and right precentral gyrus (Fig. 2B; Table 2).

### Multimodal overlapping of functional and VBM differences

On the basis of the above meta-analyses, we overlapped functional and GMV altered regions, and found decreased functional activity and increased GMV in the left insula (Fig. 2C; Table 3).

**Table 3:** Overlapping of functional and GMV differences between ASD and TD

Local Maximum			Cluster	
Region	Peak MNI coordinate (x, y, z)		No. of voxels	Breakdown (No. of voxels)
**functional activity decreased & GMV increased**				
Left insula, BA 48	-44, -14, 2		173	Left insula (100)

***Note***: GMV = gray matter volume; ASD = autism spectrum disorder; TD = typically developing; MNI = Montreal Neurological Institute; BA = Brodmann area

### Resting-state functional imaging subgroup meta-analyses

Compared with TDs, the male subgroup (11 datasets) demonstrated decreased functional activity in the right ITG (extending to the right MTG and STG), left insula (extending to the rolandic operculum and precentral gyrus), left MTG, and bilateral ACC/mPFC, and increased activity in the bilateral precuneus, and right angular gyrus (Figure S1A and Table S4). The childhood subgroup (21 datasets) demonstrated decreased functional activity in the bilateral insula (extending to the left STG and rolandic operculum), bilateral ACC/mPFC, right ITG (extending to the right MTG), left angular gyrus, and left MTG, and increased activity in the bilateral precuneus, and right SMA (Figure S1B and Table S4). The subgroup of ASD without any psychiatric comorbidities (24 datasets) showed decreased functional activity in the bilateral insula (extending to the left STG), ACC/mPFC, right MTG (extending to the right ITG), and left angular gyrus, and increased activity in the bilateral precuneus, right SMA, left cerebellum, and right fusiform gyrus (Figure S1C and Table S4). The subgroup of excluding the insufficient sample size of individuals with ASD (*n* < 20) or uncorrected for results (20 datasets) showed decreased functional activity in the left insula, bilateral ACC/mPFC, and left angular gyrus, and increased activity in the right SMA and cerebellum (Figure S1D and Table S4). In the ReHo subgroup (16 datasets), individuals with ASD displayed decreased ReHo in the bilateral insula (extending to the left STG), ACC/mPFC and left angular gyrus, and increased ReHo in the bilateral precuneus, right fusiform gyrus (extending to the right cerebellum and lingual gyrus) and SMA (Figure S1E and Table S4). There were not sufficient studies of the adolescence, adult, ALFF, fALFF, CBF to conduct subgroup meta-analyses.

### VBM Subgroup meta-analyses

Compared to TDs, the male individuals with ASD (25 datasets) displayed decreased GMV in the left inferior parietal gyri (IPG), and increased GMV in the left insula (extending to the STG and rolandic operculum), left MTG (extending to the left STG), and bilateral olfactory cortex (extending to the left striatum) (Figure S2A and Table S5). The childhood subgroup (18 datasets) demonstrated decreased GMV in the bilateral cerebellum, and right insula, and increased GMV in the left insula (extending to the left STG), bilateral cuneus cortex, and right angular gyrus (Figure S2B and Table S5). The adolescence subgroup (14 datasets) displayed reduced GMV in the bilateral ACC/mPFC (extending to the bilateral MCC), and increased GMV in the bilateral cerebellum and olfactory cortex (Figure S2C and Table S5). The adult ASD subgroup (25 datasets) displayed reduced GMV in the bilateral ACC/mPFC and precuneus, and increased GMV in the left MTG (extending to the left STG, ITG), and bilateral pre- and postcentral gyrus (Figure S2D and Table S5). The subgroup of ASD without any psychiatric comorbidities (39 datasets) showed decreased GMV in the left cerebellum and ACC/mPFC, and increased GMV in the left MTG (extending to the left insula, STG and ITG), bilateral olfactory cortex, right precentral gyrus, and left middle frontal gyrus (extending to the left superior frontal gyrus) (Figure S2E and Table S5). The subgroup of excluding the insufficient sample size of individuals with ASD (*n* < 20) or uncorrected for results (21 datasets) showed decreased GMV in the bilateral ACC, MCC, left cerebellum and superior parietal gyrus, and increased GMV in the bilateral olfactory cortex, cerebellar vermis, and left MTG (Figure S2F and Table S5).

### Sensitivity analysis and analyses of heterogeneity and publication bias

The jackknife sensitivity analyses of the pooled functional and structural meta-analysis showed high replicability and reliability (Table 2). In the functional brain activity and VBM meta-analyses, none of the brain regions showed significant heterogeneity (*I*^2^ < 50%) and publication bias (*P* > 0.05) between included studies (Table 2).

### Meta-regression analyses

In the present meta-regression analyses, the age, percentage of males and IQ were not associated with resting-state functional activity in individuals with ASD. ASD-related functional activity was negatively correlated with ADI-R score in the right ITG (Table S6).

In addition, age was positively correlated with GMV in the left MTG, and negatively correlated with GMV in the left ACC. Percentage of males, ADI-R score and IQ were not associated with GMV in individuals with ASD (Table S7).

## Discussion

To the best of our knowledge, this was the first large-scale multimodal neuroimaging meta-analysis of spontaneous functional activity and structural gray matter studies on ASD. The main findings were as follows: (1) individuals with ASD showed decreased resting-state functional activity in the left insula (extending to the left STG), bilateral ACC/mPFC, left angular gyrus, and right ITG, and increased activity in the right SMA and precuneus; (2) individuals with ASD showed decreased GMV in the ACC/mPFC and left cerebellum, and increased GMV in the left MTG (extending to the left insula and STG), bilateral olfactory cortex, and right precentral gyrus; (3) the multimodal meta-analysis showed overlapping functional and structural changes in the left insula in ASD. These findings provided a further explanation for the pathophysiological substrate of ASD.

Decreased spontaneous functional activity together with increased GMV were identified in the left insula in the present multimodal meta-analysis. The insula, a brain region that has long been underestimated, is deemed as the hub of the ‘’salience network (SN)’’, which assumes an instrumental function in social and non-social functions such as emotion, motivation, empathy, perception, risk assessment, decision-making, and sensorimotor integration [43]. Accumulating evidence shows that the insula is a critically impaired region in ASD [11, 24–26], dysfunction of which could relate to many behavioral and cognitive symptoms of ASD (e.g., mentalizing and empathizing) [8, 44, 45]. A recent experiment on an autism rat model demonstrated that cognitive impairment and repetitive/stereotypic-like behaviors might be attributable to the dysregulation of synaptic and associated receptor proteins in the insula [46]. Task-based functional meta-analyses demonstrated decreased activation in the insula in ASD during social cognitive and cognitive control [8, 10]. Previous VBM meta-analyses reported increased GMV in the insula in ASD [11, 47], especially in pediatric ASD [47]. However, recently updated VBM meta-analyses of adult ASD reported no larger GMV [26, 28]. Our subgroup analyses showed decreased functional activity and increased GMV in the insula in childhood ASD, but not in adult ASD. We speculate this might be attributed to the ‘’early brain overgrowth theory’’, which was confirmed in pediatric ASD by neuroimaging, post-mortem and head circumference development studies [24, 48–51]. More specifically, ASD is a dynamic disorder with abnormally neuronal and gray matter overgrowth in early childhood, and then further abnormal decline in adulthood [24, 52]. Evidence from the ENIGMA consortium also support this theory, indicating an increase in overall GMV in childhood and adolescence [29, 32], and normalization in adulthood in ASD [31]. The possible neurobiological mechanisms for the theory may include increased neurogenesis, decreased neuronal cell death, delayed synaptic pruning and possibly inappropriate migration of neurons [53–55]. In the autistic brain, the growth and elaboration of neural architecture and connectivity occur prematurely without being guided by functional experiences and adaptive learning; that is, the autistic brain exhibits premature “growth without guidance” in early life [55]. Therefore, our findings of decreased functional activity but increased GMV in the insula might provide additional evidence for early brain overgrowth in ASD. Intriguingly, the abnormalities in the insula were common across different psychiatric disorders and were not specific to ASD. Meta-analyses across psychiatric disorders reported common patterns of aberrant brain activation and GMV loss in the insula, which were shared across diagnoses to varying degrees, including schizophrenia (SCZ), BD, posttraumatic stress disorder (PTSD), obsessive-compulsive disorder (OCD), and MDD [56–58]. A study across six psychiatric disorders (attention-deficit/hyperactivity disorder [ADHD], ASD, BD, MDD, OCD and SCZ) from the ENIGMA consortium also indicated the transdiagnostic patterns of abnormal cortical thickness in the insula [30]. In brief, the functional and morphological evidence in the insula may suggest a key role of this region in psychiatric disorders across the lifespan (from aberrant development to degeneration).

In this study, we observed both spontaneous neural activity and GMV reduction in the ACC/mPFC in ASD. ACC/mPFC is functionally and anatomically connected to a broad set of regions engaged in social information processing, and has been recognized for its role in cognitive, behavioral and emotional functions [59–61]. Extant task-based meta-analyses indicated that the underactivation in the ACC/mPFC in ASD has been shown in a variety of tasks, including language processing, cognitive control, and so forth [8–11, 62, 63]. A cross-sectional mega-analysis of large-scale population-based samples from the ENIGMA consortium reported ASD-associated reduced structural brain asymmetry in the ACC/mPFC [64]. Numerous VBM-based meta-analyses also reported ASD-associated GMV reduction in the ACC/mPFC [10, 11, 25, 47]. Further, the subgroup analyses indicated deficits in spontaneous neural activity in the ACC/mPFC in children with ASD. In contrast, the deficits in GMV in such regions appeared in adolescents/adults with ASD. Meta-regression analyses showed that increased age correlated with lower GMV in the ACC. We, therefore, presumed the dysfunction might be more likely an early change rather than GMV reduction in the ACC/mPFC in the autistic brain. An elegant longitudinal neuroimaging study provided evidence that aberrant functional findings in 6-month-old infants could accurately predict which individuals would subsequently be diagnosed with ASD [65]. More recently, a causal link was reported between ACC/mPFC dysfunction and social behavior deficits in the Shank3 mutant rat model of ASD [66]. In the study, the selective ablation of the autism-risk gene Shank3 in ACC pyramidal neurons caused social interaction deficits while restoration of Shank3 only in ACC neurons rescued social deficits in global Shank3 mutant mice [66]. The dysfunction in the ACC/mPFC might contribute to the atypical development of intersubjectivity, joint attention and social cognition in the autistic brain [67, 68], and result in the emergence of ASD-like repetitive behaviors and social interaction deficits [69]. Developmental dysfunction has been proposed to be a key pathological change in ASD [66, 70]. Overall, the aberrant neurodevelopmental trajectory of function and structure in the ACC/mPFC is an important neurobiological feature of ASD.

Additionally, functional abnormalities were found in the DMN regions in ASD, including increased spontaneous neural activity in the right precuneus, and decreased activity in the right ITG and left angular gyrus. Previous task-based meta-analyses also indicated that individuals with ASD displayed overactivation in the precuneus [7, 11], a functional hub of the DMN [71], which broadly engaged in a variety of processing states [72]. Overactivation in the precuneus was positively correlated with social reciprocity and communication problem severity in ASD [73]. Moreover, previous meta-analytic evidence displayed resting-state functional hypoactivation in the angular gyrus in ASD [6]. The hypoactivation in the angular gyrus and ITG has been shown in a variety of task conditions [7, 8], significantly contributing to autism spectrum traits [74, 75]. Our meta-regression analyses also showed that the poorer social function (higher ADI-R score) was correlated with the lower spontaneous neural activity in the right ITG in ASD, suggesting the association of DMN dysregulation with social deficits [76]. The DMN, a large-scale functional brain network, is active in a state of rest but deactivated during task-related activities or executive function [77], and plays a key role of the engagement in processing information about the “self” and “other” [78]. Individuals with ASD struggle with both self-referential cognitive processing and inferring the mental states of others [79, 80], and dysfunctions in the DMN regarding information integration about the “self” and the “other” contribute to social deficits in ASD [78, 80]. Hence, the functional pathology of the DMN is central to social deficits in ASD, highlighting the DMN as a novel locus of dysfunction in ASD [80, 81].

Increased GMV was further detected in the left MTG in ASD in the current study. Moreover, age was positively correlated with GMV in the left MTG in ASD. Concordant with this study, several meta-analyses also reported that ASD-associated GMV increased in the MTG [11, 47, 82], which might be related to both social and language deficits in ASD [83, 84]. A mega-analysis from the ENIGMA ASD working group also found ASD-related cortical thickness alterations in the MTG [29]. Language deficits are the core feature of ASD and the failure to develop normal language comprehension is an early warning sign [85]. Considerable evidence indicated that the MTG, especially Brodmann area (BA) 21 as found in this study, might be the key brain region related to typical language impairment in ASD [85, 86]. It was reported that the normal age-related GMV and cortical thickness reductions in the MTG did not appear in individuals with ASD relative to TDs during adolescence and adulthood, which may suggest immaturation in such a region in the developmental trajectory of the autistic brain [87].

Moreover, functional and/or structural abnormalities in the primary motor cortices (i.e., SMA and precentral gyrus) and primary sensory cortices (i.e., auditory cortex and olfactory cortex) were identified in individuals with ASD in the present study. More specifically, the resting-state functional activity reductions in the left auditory cortex (STG) as well as increases in the right SMA, and GMV increases in the left STG, bilateral olfactory cortex and right precentral gyrus were found in this study. In line with our findings, the existing task-based and task-free meta-analyses indicated hyperactivation in the SMA [6, 8], and VBM-based meta-analyses reported GMV increases in the precentral gyrus [10, 82]. Previous studies proposed that motor deficits (e.g., repetitive and stereotyped behavior) represented a potential core feature of ASD [88], and might directly limit social communication opportunities and subsequently impairs social development [88]. The SMA and precentral gyrus are typically associated with motor performance [8, 86, 89], and the functional and anatomical alterations in these regions may result in abnormal motor activity in populations with ASD [89]. ASD is also characterized by atypical sensory functioning in the visual, tactile, auditory and even olfactory systems [90]. Previously published meta-analyses indicated reduced spontaneous neural activity and increased GMV in the STG in ASD [6, 7, 9, 11, 47]. The STG was associated with typically impaired auditory processing including language in ASD [91, 92], and it was pointed out STG abnormalities were related to the regression of language ability [93, 94]. In addition, impaired odor perception and central olfactory processing were reported in individuals with ASD [90, 95]. A recent machine learning study indicated that the volumetric abnormalities in the olfactory cortex were the key to the identification of ASD [96]. The olfactory alterations have been suggested as a marker for diagnosing ASD or distinguishing ASD from other developmental disorders [96, 97]. In summary, given extant evidence, primary motor and sensory abnormalities of function and structure regularly present and may not be neglectable physiological factors underlying social impairments in ASD.

In terms of the cerebellar alterations in ASD, our findings reported GMV reductions in the left cerebellar posterior lobe, which were in line with the findings of previous VBM meta-analyses [11, 27, 47]. More recently, sufficient evidence pointed out that the cerebellar posterior lobe was regarded as the cognitive/limbic cerebellum and mainly participated in cognition and emotional regulation, while the sensorimotor cerebellum was presented in the anterior lobe [98, 99]. Damage to or reduced volume of the cerebellum was associated with high rates of ASD [99]. Cerebellar posterior lobe lesions resulted in autistic-like behaviors (e.g., abnormal social interaction, and repetitive and stereotyped behavior) [98, 99]. Thus, the cerebellum might also be involved in the pathophysiological mechanism of ASD.

This study had certain limitations. Firstly, an inevitable methodological limitation of meta-analysis is the failure to obtain individual study data, which obstructs the examination of individual differences. Secondly, diversified diagnostic tools, imaging characteristics, imaging parameters, statistical thresholds, multiple comparison corrections and post-hoc corrections were used in included studies, influencing the results to some extent. Thirdly, the relationships between different local phenomena of resting-state physiology are not completely clear; hence it may not be convincing to characterize local resting-state function by the combination of different functional modalities. Additionally, a significant proportion (74%) of individuals with ASD had at least one co-occurring psychiatric comorbidity [100, 101]. In current meta-analysis, studies of ASD with psychiatric comorbidities were not excluded. Nevertheless, pure subgroup analyses of ASD without psychiatric comorbidities were conducted. Furthermore, although subgroup analyses were conducted, certain age differences between functional and structural meta-analyses may somewhat influence the results. Finally, an inherent concern is the influence of childhood experiences (e.g., maltreatment, bullying, and other adverse childhood experiences) and environmental factors (e.g., air pollution, maternal diseases, and infections) on the neurofunctional and neuroanatomical changes, when discussing the neural basis of any childhood-onset disorder [102]. To minimize interference in this respect, more homogenous samples might benefit future further studies on neurobiological correlates associated with ASD.

## Conclusions

In conclusion, the current multimodal meta-analysis demonstrated that ASD exhibited similar alterations in both function and structure of the insula and ACC/mPFC. In addition, the DMN, primary motor and sensory regions exhibited functional or structural alterations in ASD. In general, our findings provided valuable insights into the underlying pathophysiology of ASD.

### Electronic supplementary material

Below is the link to the electronic supplementary material.

Supplementary Material 1

## Data Availability

All data generated or analyzed during this study are available in those included published articles and its supplementary information files.
