# Supplementary material for: Systematic review and meta-analysis: multimodal functional and anatomical neural alterations in autism spectrum disorder
Source: Mol Autism. 2024 Apr 4;15:16. doi: 10.1186/s13229-024-00593-6 (PMC10996269; doi:10.1186/s13229-024-00593-6)
Supplement: Supplementary file 1 — Supplementary Material 1 [file 13229_2024_593_MOESM1_ESM.docx]

***Supplementary Material***

**(Systematic Review and Meta-analysis: Multimodal Functional and Anatomical Neural Alterations in Autism Spectrum Disorder)**

**Method**

**Main Meta-analysis** **of Structural Differences**

Meta-analysis of structural (gray matter volume [GMV]) group differences was conducted using the Seed-based d Mapping (SDM) software ([www.sdmproject.com](http://www.sdmproject.com)). Briefly, we used the reported peak coordinates and their statistics (e.g., t-values) to recreate an effect-size signed map of the difference between individuals with autism spectrum disorder (ASD) and typically developing individuals (TDs) for each original study in SDM software (Radua et al., 2012; W. Wang et al., 2018). We selected the "VBM - gray matter" modality and the "gray matter" correlation template within the "gray matter" mask to improve the accuracy of effect size maps. Next, the maps were then consolidated in a standard random-effects model weighing sample size (i.e., studies with larger sample size or lower variability contribute more), intra-study variability, and between-study heterogeneity, and multiple imputations are pooled according to Rubin’s rules.(Albajes-Eizagirre, Solanes, Vieta, & Radua, 2019) According to the recommendations of the developer of SDM and previous studies, an uncorrected p < 0.005 threshold with minimum cluster extent >50 voxels and SDM-Z > 1 was used to reduce the possibility of false positive results and to optimally balance false positives and negatives (Radua et al., 2012; W. Wang et al., 2018). In addition, MRIcron software package (www.mricro.com/mricron/) was used to visualize SDM maps.

**Figure S1 Subgroup meta-analyses results regarding functional differences between ASD and TD**

**
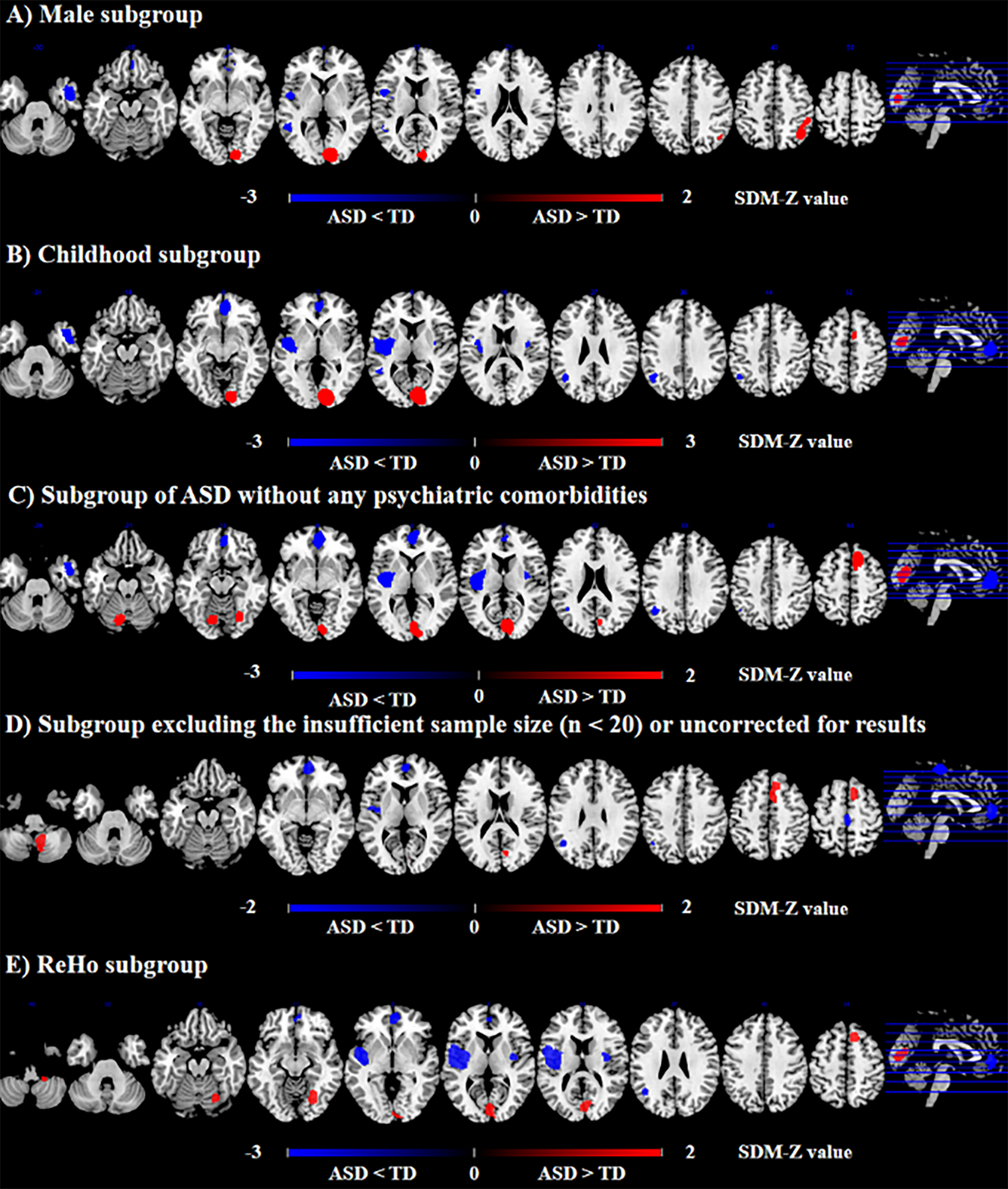
**

***Note:*** A) male subgroup; B) childhood (0-12 years old) subgroup; C) subgroup of ASD without any psychiatric comorbidities; D) subgroup excluding the insufficient sample size of individuals with ASD (n < 20) or uncorrected for results; E) ReHo subgroup. Areas with increased resting-state functional activity value are displayed in red, and areas with decreased resting-state functional activity value are displayed in blue. The color bar indicates the maximum and minimum SDM-Z values. ASD = autism spectrum disorder; TD = typically developing; SDM = seed-based d mapping; ReHo = regional homogeneity.

**Figure S2 Subgroup meta-analyses results regarding GMV differences between ASD and TD**

**
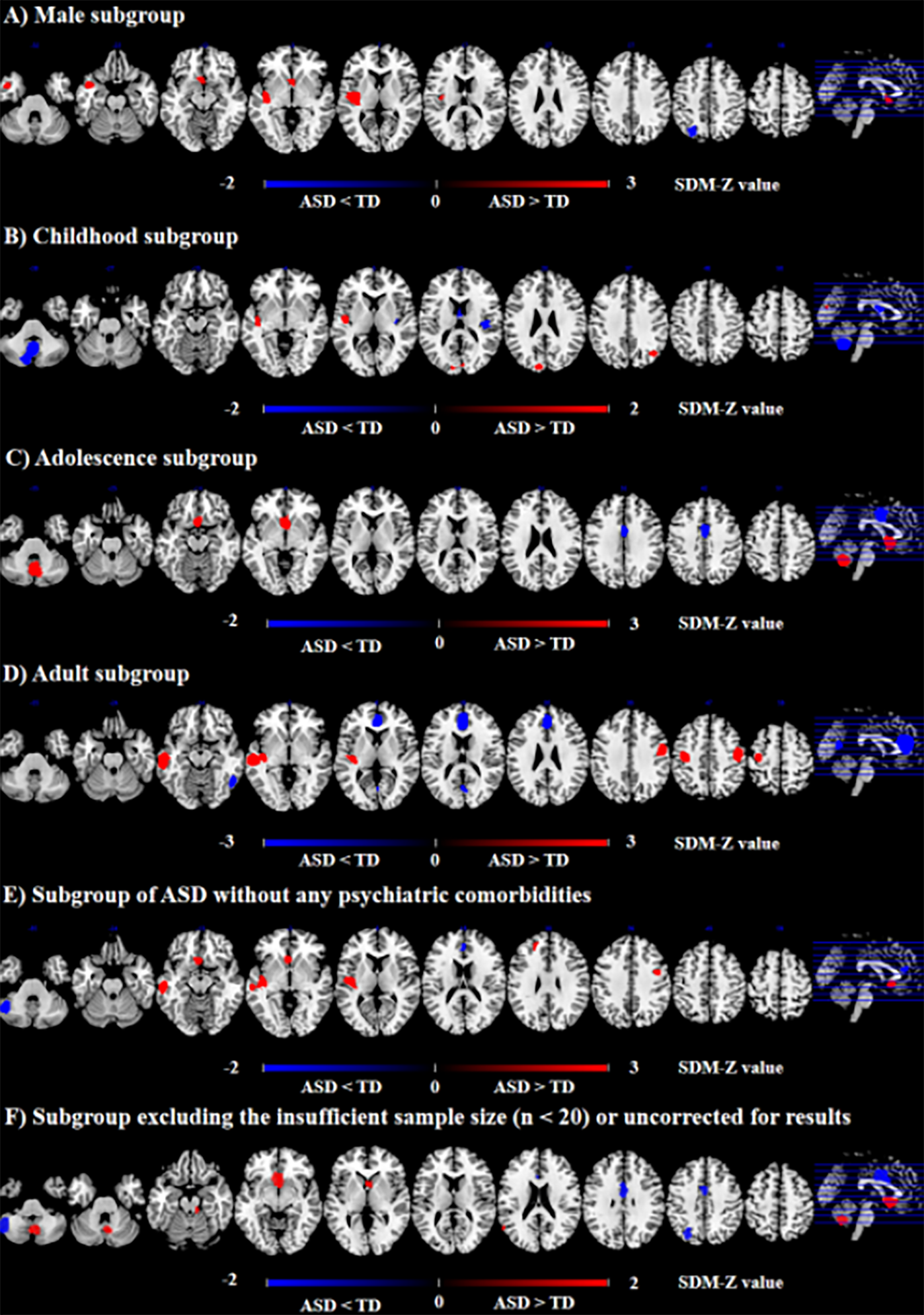
**

***Note:*** A) male subgroup; B) childhood (0-12 years old) subgroup; C) adolescence (13-18 years old) subgroup; D) adult (age > 18) subgroup; E) subgroup of ASD without any psychiatric comorbidities; F) subgroup excluding the insufficient sample size of individuals with ASD (n < 20) or uncorrected for results. Areas with increased GMV value are displayed in red, and areas with decreased GMV value are displayed in blue. The color bar indicates the maximum and minimum SDM-Z values. ASD = autism spectrum disorder; TD = typically developing; SDM = seed-based d mapping; GMV = gray matter volume.

**Table S1 Quality assessment checklist (score 0/0.5/1 per item; total score out of 10) ***

| Category 1: Participants |
| --- |
| 1. Patients were evaluated prospectively, specific diagnostic criteria were applied, and demographic data were reported. |
| 2. Healthy comparison participants were evaluated prospectively, psychiatric and medical illnesses were excluded. |
| 3. Important variables (e.g., age, sex, illness duration, onset, medication status, comorbidity, severity of illness) were checked either by stratification or statistically. |
| 4. Sample size per group > 10. |
| Category 2: Methods for image acquisition and analysis |
| 5. Whole brain analysis was automated with no a priori regional selection. |
| 6. Coordinates reported in a standard space. |
| 7. The imaging technique used was clearly described so that it could be reproduced. |
| 8. Measurements were clearly described so that they could be reproduced. |
| Category 3: Results and conclusions |
| 9. Statistical parameters for significant and important nonsignificant differences were provided. |
| 10. Conclusions were consistent with the results obtained and the limitations were discussed. |
| *When criteria were partially met, 0.5 points were awarded. |

**Table S2 Demographic, clinical and imaging characteristics of the included studies of resting-state functional activity**

| **study** | **ASD** **individuals** | | | | **TDs** | | | **Imaging characteristics** | | | | | **Diagnostic tool** | **Quality**  **score** |
| --- | --- | --- | --- | --- | --- | --- | --- | --- | --- | --- | --- | --- | --- | --- |
|  | **Participants(male)** | **Age(M±SD)** | **IQ(M ±SD)** | **ADI-R** | **Participants(male)** | **Age(M±SD)** | **IQ(M±SD)** | **Modality/analysis** | **Scanner field strength** | **Software** | **FWHM** | **Threshold** |  |  |
| (Du L, 2021) | 26(20) | 10.4±1.4 | 89.3±7.9 | NA | 24(19) | 10.6±1.1 | 92.8±6.5 | rs-fMRI/ALFF/ReHo | 3.0 T | SPM8 | NA | P < 0.05 (AlphaSim corrected) | DSM-4 | 10.0 |
| (Cheng H et al., 2016) | 33(33) | 4.0±1.0 | NA | 48.0±9.0 | 26(26) | 4.0±1.0 | NA | rs-fMRI/ALFF/fALFF | 3.0 T | SPM5 | 6mm | P < 0.05 (AlphaSim corrected) | DSM-4 | 9.5 |
| (Cao, Zhang, Liu, & Yang, 2016) | 12(10) | 3.9±0.9 | NA | NA | 12(5) | 5.6±2.1 | NA | rs-fMRI/ALFF/ReHo | 3.0 T | DPARSF | 4mm | P < 0.001 (AlphaSim corrected) | DSM-5 | 9.5 |
|  | 20(18) | 4.2±1.3 | NA | NA | 14(5) | 5.4±2.0 | NA | ASL-fMRI/CBF | 3.0 T | Cereflow | 4mm | P < 0.05 (uncorrected) | DSM-5 | 9.0 |
| (Li, Rossbach, Jiang, & Du, 2018) | 15(15) | 8.9±3.1 | 50.5±11.3 | NA | 15(15) | 10.5±2.6 | 127.3±13.8 | rs-fMRI/ALFF/ReHo | 3.0 T | REST | 4mm | P < 0.005 (AlphaSim corrected) | DSM-5 | 10.0 |
| (Mei et al., 2022) | 65(56) | 13.3±5.0 | 103.0±17.0 | NA | 55(40) | 14.5±6.0 | 115.0±11.0 | rs-fMRI/ALFF | 3.0 T | SPM | 4mm | P < 0.05 (TFCE corrected) | DSM-4 | 10.0 |
| (Yue et al., 2022) | 34(34) | 9.3±1.2 | 101.3±17.0 | NA | 49(49) | 9.4±1.1 | 107.6±11.8 | rs-fMRI/ALFF/ReHo | 3.0 T | REST | 6mm | P < 0.05 (FDR corrected) | DSM-4 | 10.0 |
| (Itahashi et al., 2015) | 50(43) | 30.8±7.4 | 105.6±14.1 | NA | 50(43) | 31.6±7.6 | 108.1±9.0 | rs-fMRI/fALFF | 1.5 T | REST | 6mm | P < 0.05 (GRF corrected) | DSM-4 | 10.0 |
| (Qiu XY, 2009) | 23(20) | 5.1±2.3 | NA | NA | 10(8) | NA | NA | SPECT/CBF | NA | SPM5 | 8mm | NA | DSM-4 | 9.0 |
| (Xiu LJ, 2010) | 23(20) | 7.2±3.0 | NA | NA | 8(7) | 5.5±2.4 | NA | SPECT/CBF | NA | SPM5 | 20mm | P < 0.001 (uncorrected) | ICD-10 | 9.0 |
| (Pagani et al., 2012) | 13(7) | 31.8±8.6 | 104.2±17.1 | NA | 10(5) | 28.5±7.5 | 115.7±10.8 | PET/CBF | NA | SPM2 | 8mm | P < 0.001 (corrected) | ADOS-G and RAADS-R | 9.5 |
| (Jann et al., 2015) | 17(13) | 13.8±2.0 | 107.8±18.7 | 40.9±10.4 | 22(19) | 12.8±3.6 | 107.8±14.3 | ASL-fMRI/CBF | 3.0 T | NA | 8mm | P < 0.05 (AlphaSim corrected) | ADI-R and ADOS | 9.5 |
| (Yerys et al., 2018) | 33(33) | 14.9±1.7 | 94.0±20.0 | NA | 25(25) | 14.9±1.7 | 123±18.0 | ASL-fMRI/CBF | 3.0 T | FSL | 8mm | P < 0.05 (FDR corrected) | DSM-4 | 10.0 |
| (Hao GF, 2020) | 15(15) | 3.8±0.9 | 82.4±7.2 | NA | 15(15) | 4.1±1.1 | 86.6±4.8 | rs-fMRI/ReHo | 3.0 T | REST | 4mm | P < 0.05 (AlphaSim corrected) | DSM-5 | 9.5 |
| (Jao Keehn et al., 2019) | 57(47) | 13.8±2.6 | 104.4±17.2 | 37.9±12.2 | 51(42) | 13.2±2.7 | 106.4±10.7 | rs-fMRI/ReHo | 3.0 T | AFNI | 6mm | P < 0.05 (corrected) | ADI-R, ADOS and DSM-5 | 9.5 |
| (Lan et al., 2021) | 86(86) | 4.1±1.0 | NA | NA | 54(54) | 3.9±1.0 | NA | rs-fMRI/ReHo | 3.0 T | REST | 8mm | P < 0.05 (FDR corrected) | CARS, ABC and DSM-5 | 9.5 |
| (Li X et al., 2013) | 16(13) | 12.0±3.0 | 83.9±15.5 | NA | 16(13) | 12.0±3.0 | 91.1±12.4 | rs-fMRI/ReHo | 3.0 T | REST | 4mm | P < 0.05 (uncorrected) | ADI-R and DSM-4 | 9.0 |
| (Li Y, Zhang L, Xu H, & Jiang Q, 2022) | 45(29) | 6.7±0.8 | NA | NA | 25(18) | 6.5±0.8 | NA | rs-fMRI/ReHo | 3.0 T | DPARSF | NA | P < 0.001 (AlphaSim corrected) | DSM-5 | 9.5 |
| (Maximo, Keown, Nair, & Muller, 2013) | 29(25) | 13.8±2.4 | 107.9±19.0 | 38.1±14.5 | 29(22) | 13.5±2.2 | 108.0±8.9 | rs-fMRI/ReHo | 3.0 T | AFNI | 6mm | P < 0.05 (corrected) | ADI-R and ADOS | 10.0 |
| (Paakki et al., 2010) | 28(20) | 14.6±1.6 | NA | NA | 27(18) | 14.5±1.5 | NA | rs-fMRI/ReHo | 1.5 T | AFNI | 4mm | P < 0.05 (AlphaSim corrected) | ICD-10 | 8.5 |
| (Wang LL, 2016) | 20(16) | 3.4±1.1 | NA | NA | 20(15) | 3.3±1.1 | NA | rs-fMRI/ReHo | 3.0 T | REST | 6mm | P < 0.01 (AlphaSim corrected) | DSM-4 | 9.0 |
| (Wang Y, 2018) | 39(34) | 9.6±2.7 | 101.5±17.4 | NA | 47(37) | 10.2±2.7 | 102.0±16.7 | rs-fMRI/ReHo | 3.0 T | SPM12 | 4mm | P < 0.05 (FDR corrected) | DSM-5 | 9.5 |
| (Yin TN et al., 2021) | 39(34) | 13.3±3.0 | 97. 8±25. 7 | NA | 43(37) | 13.3±3.0 | 106. 1±14. 2 | rs-fMRI/ReHo | 3.0 T | DPARSF | 4mm | P < 0.05 (GRF corrected) | DSM-5 | 9.5 |
| (Zhao, Zhu, Cao, Cheng, Lin, Sun, Li, et al., 2022) | 48(39) | 13.0±1.9 | 106.9±19.2 | NA | 63(43) | 12.9±1.8 | 111.0±14.3 | rs-fMRI/ReHo | 3.0 T | DPABI | 4mm | P < 0.001 (FWE corrected) | DSM-5 | 10.0 |

***Note:*** ASD = autism spectrum disorder; TD = typically developing; SD = standard deviation; IQ = intelligence quotient; FWHM = full width at half maximum; NA = not available; ALFF = amplitude of low-frequency fluctuation; fALFF = fractional amplitude of low-frequency fluctuation; ReHo = regional homogeneity; CBF = cerebral blood flow; ASL = arterial spin labelling; SPECT = single-photon emission computed tomography; PET = positron emission tomography; SPM = statistical parametric mapping; DPABI = Data Processing & Analysis for Brain Imaging; DPARSF = data processing assistant for resting-State fMRI software; REST = Resting State fMRI Data Analysis Toolkit; ANFI = Analysis of Functional NeuroImages; FWE = family wise error; FDR = false discovery rate; GRF = Gaussian random field; TFCE = the Threshold-Free Cluster Enhancement; DSM = Diagnostic and Statistical Manual of Mental Disorders; ICBM = International Consortium for Brain Mapping; ADI-R = Autism Diagnostic Interview-Revised; ADOS = Autism Diagnostic Observation Schedule; ABC = Autism Behavior Checklist; RAADS-R = Ritvo Autism and Asperger Diagnostic Scale Revised; ICD = international Classification of diseases.

**Table S3 Demographic, clinical and imaging characteristics of the included studies of VBM**

| **study** | **ASD individuals** | | | | **TDs** | | | **Imaging characteristics** | | | | | **Diagnostic tool** | **Quality**  **score** |
| --- | --- | --- | --- | --- | --- | --- | --- | --- | --- | --- | --- | --- | --- | --- |
|  | **Participants(male)** | **Age(M±SD)** | **IQ(M ±SD)** | **ADI-R** | **Participants(male)** | **Age(M±SD)** | **IQ(M±SD)** | **Modality/analysis** | **Scanner field strength** | **Software** | **FWHM** | **Threshold** |  |  |
| (Abell et al., 1999) | 15(12) | 28.8±6.6 | NA | NA | 15(12) | 25.3±3.1 | NA | VBM/gray matter volume | 2.0 T | SPM96 | 12mm | P < 0.001 (uncorrected) | DSM-4 | 9.0 |
| (Anteraper et al., 2020) | 28(24) | 24.7 ±5.2 | 105.9 ±16.0 | NA | 38(25) | 24.3±3.6 | 118.3 ±11.0 | VBM/gray matter volume | 3.0 T | SPM12 | 6mm | P < 0.05 (FDR corrected) | DSM-4 | 10.0 |
| (Craig et al., 2007) | 14(0) | 37.9±11.4 | 103.4±17.0 | NA | 19(0) | 35.0±14.0 | 111.2±14.5 | VBM/gray matter volume | 1.5 T | SPM2 | 5mm | P < 0.05 (uncorrected) | ICD-10, ADI-R and ADOS | 9.5 |
| (David et al., 2014) | 15(7) | 33.2±7.4 | NA | NA | 12(7) | 32.9±7.6 | 111.4±15.1 | VBM/gray matter volume | 3.0 T | SPM8 | 12mm | P < 0.001 (uncorrected) | ADOS | 9.0 |
| (Ecker et al., 2010) | 22(22) | 27.0 ±7.0 | 104.0±15.0 | 35.0±12.0 | 22(22) | 28.0±7.0 | 111.0±10.0 | VBM/gray matter volume | 3.0 T | SPM5 | 8mm | P < 0.1 (uncorrected) | ICD-10, ADI-R and ADOS | 9.5 |
| (Ecker et al., 2012) | 89(89) | 26.0±7.0 | 110.0±15.0 | 37.0±9.0 | 89(89) | 28.0±6.0 | 113.0±12.0 | VBM/gray matter volume | 3.0 T | CamBA | 4mm | P < 0.004 (uncorrected) | ICD-10 and ADI-R | 9.5 |
| (Katz et al., 2016) | 23(23) | 26.7±6.5 | 105.2±18.6 | NA | 32(32) | 29.8±9.2 | 114.5±10.2 | VBM/gray matter volume | 3.0 T | R software | 7mm | P < 0.05 (FDR corrected) | DSM-4, ADI-R and ADOS | 9.5 |
| (Kojima et al., 2019) | 39(39) | 29.9±6.8 | 107.5±11.5 | 31.0±11.9 | 39(39) | 31.5±4.5 | 109.8±8.1 | VBM/gray matter volume | 3.0 T | FreeSurfer | 10mm | P < 0.05 (uncorrected) | DSM-4, ADI-R and ADOS | 9.5 |
| (Kosaka et al., 2010) | 32(32) | 23.8±4.2 | 101.6±15.6 | NA | 40(40) | 22.5±4.3 | 109.7±7.9 | VBM/gray matter volume | 3.0 T | SPM5 | 8mm | P < 0.05 (FDR corrected) | DSM-4 and DISCO | 10.0 |
| (Lai et al., 2013) | 60(30) | 27.5±7.5 | 115.2±13.9 | NA | 60(30) | 27.9±6.1 | 118.9±9.4 | VBM/gray matter volume | 3.0 T | SPM8 | 4mm | P < 0.05 (FDR corrected) | DSM-4 | 10.0 |
| (Lin, Tseng, Lai, Chang, & Gau, 2017) | 18(18) | 13.3±2.5 | 103.8±17.8 | NA | 48(48) | 12.8±2.6 | 112.5±11.6 | VBM/gray matter volume | 3.0 T | SPM8 | 4mm | P < 0.05 (FWE corrected) | DSM-4 | 10.0 |
| (McAlonan et al., 2002) | 21(19) | 32.0±10.0 | 96.0±15.0 | NA | 24(22) | 33.0±7.0 | 114.0±14.0 | VBM/gray matter volume | 1.5 T | NA | NA | P < 0.002 (uncorrected) | ICD-10 | 8.5 |
| (Mueller et al., 2013) | 12(9) | 35.5±11.4 | 111.3±13.4 | NA | 12(8) | 33.3±9.0 | 110.8±14.4 | VBM/gray matter volume | 3.0 T | FSL | 5mm | P < 0.001 (uncorrected) | ICD-10 | 9.0 |
| (Sato et al., 2017) | 36(25) | 27.0 ±8.0 | 110.4±13.3 | NA | 36(25) | 24.9±5.5 | NA | VBM/gray matter volume | 3.0 T | SPM8 | 8mm | P < 0.05 (FDR corrected) | DSM-5 | 9.0 |
| (Schmitz et al., 2006) | 10(10) | 38.0±9.0 | 105.0±14.0 | NA | 12(12) | 39.0±9.0 | 106.0±13.0 | VBM/gray matter volume | 3.0 T | SPM99 | 10mm | P < 0.001 (uncorrected) | ICD-10 | 8.5 |
| (Toal et al., 2009) | 30(30) | 30.5±10.0 | 91.0±14.0 | NA | 16(16) | 36.0±10.0 | 99.0±9.0 | VBM/gray matter volume | 1.5 T | SPM5 | 5mm | P < 0.05 (uncorrected) | ICD-10 and ADI-R | 9.0 |
| (Toal et al., 2010) | 65(57) | 31.0±10.0 | 98.0±21.0 | NA | 33(30) | 32.0±9.0 | 105.0±12.0 | VBM/gray matter volume | 1.5 T | SPM2 | 8mm | P < 0.05 (uncorrected) | ICD-10, ADI-R and ADOS | 9.0 |
| (J. Wang et al., 2017) | 31(31) | 4.8±1.1 | NA | 44.7±9.9 | 31(31) | 4.8±0.9 | NA | VBM/gray matter volume | 3.0 T | SPM12 | 6mm | P < 0.05 (AlphaSim corrected) | DSM-5, ADI-R and ADOS | 9.5 |
| (Wilson, Tregellas, Hagerman, Rogers, & Rojas, 2009) | 10(8) | 30.1±9.2 | 91.5±19.7 | NA | 10(8) | 29.4±7.9 | 127.2±9.0 | VBM/gray matter volume | 1.5 T | SPM2 | 12mm | P < 0.05 (FDR corrected) | DSM-4, ADI and ADOS | 9.0 |
| (Cai et al., 2018) | 38(32) | 9.6±3.4 | 75.8±25.1 | 42.8±18.1 | 27(26) | 8.3±2.3 | 98.6±16.6 | VBM/gray matter volume | 3.0 T | SPM8 | 8mm | P < 0.001 (uncorrected) | DSM-5, ADI-R and ADOS-G | 9.5 |
| (Bonilha et al., 2008) | 12(12) | 12.4±4 | NA | NA | 16(16) | 13.2±5.0 | NA | VBM/gray matter volume | 2.0 T | SPM5 | 10mm | P < 0.05 (FDR corrected) | DSM-4 and ICD-10 | 9.0 |
| (Freitag et al., 2008) | 15(13) | 17.6±3.6 | 101.2±21.2 | 25.8±11.6 | 15(13) | 18.6±1.2 | 112.1±18 | VBM/gray matter volume | 1.5 T | SPM99 | 12mm | P < 0.001 (uncorrected) | DSM-5, ADI-R and ADOS-G | 9.0 |
| (Greimel et al., 2013) | 47(47) | 21.4±10.1 | 107.5±16.6 | NA | 51(51) | 18.3±7.5 | 112.5±12.4 | VBM/gray matter volume | 1.5 T | SPM5 | 8mm | P < 0.001 (uncorrected) | DSM-5, ADI-R and ADOS-G, ICD-10 | 9.0 |
| (Lim et al., 2015) | 19(19) | 14.9±1.9 | 113.0±15.7 | 35.4±11.2 | 33(33) | 14.3±2.5 | 110.0±11.5 | VBM/gray matter volume | 3.0 T | SPM8 | 8mm | P < 0.05 (FWE corrected) | ICD-10 and ADOS | 10.0 |
| (McAlonan et al., 2008) | 17(14) | 11.4±2.5 | NA | 39.3±11.0 | 55(47) | 10.7±2.7 | NA | VBM/gray matter volume | 1.5 T | BAMM software | NA | P < 0.002 (FWE corrected) | DSM-4, ADI-R | 8.5 |
|  | 16(13) | 11.7±2.8 | NA | 39.7±10.5 | 55(47) | 10.7±2.7 | NA | VBM/gray matter volume | 1.5 T | BAMM software | NA | P < 0.002 (FWE corrected) | DSM-4, ADI-R | 8.5 |
| (Ni et al., 2018) | 53(53) | 13.0±2.0 | 108.3±15.1 | 39.3±14.4 | 61(61) | 12.4±2.4 | 112.0±10.9 | VBM/gray matter volume | 3.0 T | SPM8 | 4mm | P < 0.05 (FWE corrected) | DSM-4 and ICD-10 | 9.5 |
|  | 28(28) | 12.0±2.2 | 105.6±12.0 | 43.2±14.4 | 61(61) | 12.4±2.4 | 112.0±10.9 | VBM/gray matter volume | 3.0 T | SPM8 | 4mm | P < 0.05 (FWE corrected) | DSM-4 and ICD-10 | 9.5 |
| (Retico et al., 2016) | 76(38) | 4.4±1.4 | 71.0±22.0 | NA | 76(38) | 4.4±1.5 | NA | VBM/gray matter volume | 1.5 T | SPM8 | 8mm | P < 0.001 (uncorrected) | DSM-4, ADOS | 9.0 |
| (Riedel et al., 2014) | 30(19) | 35.4±9.1 | 124.5±12.3 | NA | 30(19) | 35.5±8.3 | 123.6±13.8 | VBM/gray matter volume | 3.0 T | SPM8 | 8mm | P < 0.05 (FWE corrected) | DSM-4 and ICD-10 | 9.5 |
| (Rojas et al., 2006) | 24(24) | 20.8±10.6 | 94.8±20.6 | NA | 23(23) | 21.4±10.9 | 118.7±11.2 | VBM/gray matter volume | 1.5 T | SPM2 | 8mm | P < 0.05 (FDR corrected) | DSM-4, ADI and ADOS | 10.0 |
| (Poulin-Lord et al., 2014) | 23(20) | 19.8±4.7 | 100.3±10.5 | 44.5±21 | 22(19) | 22.6±5.6 | 107.3±12.5 | VBM/gray matter volume | 3.0 T | SPM8 | 10mm | P < 0.05 (FWE corrected) | DSM-5, ADI-R and ADOS-G | 10.0 |
| (Radeloff et al., 2014) | 34(31) | 19.1±5.1 | 105.7±12.9 | 41.9±13.1 | 26(22) | 19.5±3.5 | 107.8±12.0 | VBM/gray matter volume | 3.0 T | SPM8 | 8mm | P < 0.001 (uncorrected) | ICD-10, ADI-R and ADOS | 9.5 |
| (Waiter et al., 2004) | 16(16) | 15.4±2.2 | 100.4±21.7 | NA | 16(16) | 15.5±1.6 | 99.7±18.3 | VBM/gray matter volume | 1.5 T | SPM2 | 8mm | P < 0.001 (uncorrected) | DSM-4, ADI-R and ADOS-G | 9.5 |
| (Yang et al., 2018) | 16(10) | 10.4±2.8 | 45.3±1.0 | NA | 16(10) | 10.5±3.1 | 97.5±5.9 | VBM/gray matter volume | 3.0 T | FSL | 3mm | P < 0.001 (uncorrected) | DSM-4 | 9.5 |
| (Foster et al., 2015) | 38(38) | 12.4±2.9 | 102.5±17 | NA | 46(46) | 12.6±3.0 | 113.1±12.0 | VBM/gray matter volume | 3.0 T | SurfStat | 8mm | P < 0.001 (uncorrected) | DSM-4 and ADOS | 8.5 |
| (Poustka et al., 2012) | 18(16) | 9.7±2.1 | 111±14.4 | 36.7±13.9 | 18(16) | 9.7±1.9 | 112.8±14.9 | VBM/gray matter volume | 1.5 T | SPM5 | 10mm | P < 0.05 (FWE corrected) | ADI-R and ADOS | 9.5 |
| (Kurth et al., 2011) | 52(38) | 11.2±4.0 | 102.2±16.1 | NA | 52(38) | 11.1±3.6 | 106±13.3 | VBM/gray matter volume | 1.5 T | SPM8 | 8mm | P < 0.05 (FWE corrected) | DSM-4, ADI-R and ADOS | 9.5 |
| (Kaufmann et al., 2013) | 10(8) | 14.7±5.0 | 102.3±15.9 | 34.7±11.5 | 10(8) | 13.8±5.3 | 109.5±6.4 | VBM/gray matter volume | 1.5 T | SPM8 | 6mm | P < 0.001 (uncorrected) | DSM-4, ADI-R and ADOS | 8.5 |
| (Hyde, Samson, Evans, & Mottron, 2010) | 15(15) | 22.7±6.4 | 100.4±12.6 | 49.0±11.0 | 13(13) | 19.2±5.0 | 106.6±12.1 | VBM/gray matter volume | 3.0 T | CIVET | 12mm | P < 0.05 (FDR corrected) | ADI-R and ADOS-G | 9.5 |
| (Cheng, Chou, Fan, & Lin, 2011) | 25(25) | 13.7±2.5 | 101.6±18.9 | 47.4±8.8 | 25(25) | 13.5±2.1 | 109±9.5 | VBM/gray matter volume | 1.5 T | SPM2 | 8mm | P < 0.05 (FWE corrected) | DSM-4, ADI-R | 10.0 |
| (Brieber et al., 2007) | 15(15) | 14.2±1.9 | 106.8±21.4 | 42.8±17.7 | 15(15) | 13.3±1.8 | 107.7±12.7 | VBM/gray matter volume | 1.5 T | SPM2 | 12mm | P < 0.001 (uncorrected) | DSM-4, ADI-R, ADOS-G, and ICD-10 | 9.5 |
| (Albajara Saenz et al., 2020) | 18(16) | 10.4±1.4 | 100.2±16.7 | NA | 17(12) | 10.5±1.6 | 118.6±14.9 | VBM/gray matter volume | 3.0 T | SPM12 | 5mm | P < 0.05 (TFCE corrected) | DSM-5 | 10.0 |
| (Guo et al., 2021) | 67(67) | 5.0±1.2 | NA | 42.8±10.0 | 63(63) | 5.2±0.9 | NA | VBM/gray matter volume | 3.0 T | SPM12 | 8mm | P < 0.05 (AlphaSim corrected) | DSM-5, | 9.5 |
| (Li et al., 2019) | 16(16) | 8.8±3.1 | 50.1±10.8 | NA | 16(16) | 10.8±2.9 | 124.5±15.7 | VBM/gray matter volume | 3.0 T | REST | 8mm | P < 0.001 (AlphaSim corrected) | DSM-5 | 10.0 |
| (Noppari et al., 2022) | 20(20) | 28.0±6.0 | NA | NA | 19(19) | 29.0±8.0 | NA | VBM/gray matter volume | 3.0 T | SPM12 | 8mm | P < 0.05 (FDR corrected) | DSM-5 | 9.5 |
| (Seng, Lai, Goh, Tseng, & Gau, 2022) | 70(66) | 15.8±3.4 | 99.2±18.3 | NA | 124(114) | 16.0±3.7 | 109.7±11.8 | VBM/gray matter volume | 3.0 T | SPM12 | 4mm | P < 0.05 (FDR corrected) | DSM-4 | 10.0 |
|  | 70(66) | 15.5±3.1 | 102.9±16.7 | NA | 124(114) | 16.0±3.7 | 109.7±11.8 | VBM/gray matter volume | 3.0 T | SPM12 | 4mm | P < 0.05 (FDR corrected) | DSM-4 | 10.0 |
| (H. Wang et al., 2022) | 24(21) | 9.0±2.0 | 102.4±19.6 | NA | 19(11) | 9.0±1.7 | 109.7±12.9 | VBM/gray matter volume | 3.0 T | SPM8 | 6mm | P < 0.001 (GRF corrected) | DSM-4 | 9.5 |
|  | 18(15) | 14.1±1.2 | 108.7±13.1 | NA | 18(14) | 14.7±2.1 | 116.5±10.0 | VBM/gray matter volume | 3.0 T | SPM8 | 6mm | P < 0.001 (GRF corrected) | DSM-4 | 9.5 |
|  | 10(9) | 21.9±3.0 | 115.1±17.9 | NA | 13(12) | 22.6±3.8 | 121.5±5.3 | VBM/gray matter volume | 3.0 T | SPM8 | 6mm | P < 0.001 (GRF corrected) | DSM-4 | 9.0 |
| (Zhao, Zhu, Cao, Cheng, Lin, Sun, Jiang, et al., 2022) | 48(39) | 13.0±1.9 | 106.9±19.2 | NA | 63(43) | 12.9±1.8 | 111.0±14.3 | VBM/gray matter volume | 3.0 T | SPM | 8mm | P < 0.001 (FWE corrected) | DSM-5 | 10.0 |
| (Lin, Ni, Lai, Tseng, & Gau, 2015) | 86(86) | 15.0±4.6 | 102.9±16.9 | 42.6±12.8 | 90(90) | 15.7±6.0 | 114.4±10.7 | VBM/gray matter volume | 3.0 T | SPM8 | 4mm | P < 0.05 (FWE corrected) | DSM-4 | 9.5 |
| (Riva et al., 2013) | 26(23) | 5.9±2.5 | 51.6±9.3 | NA | 21(13) | 6.8±2.1 | NA | VBM/gray matter volume | 1.5 T | SPM8 | 8mm | P < 0.05 (FDR corrected) | DSM-4 | 9.0 |
| (Ke et al., 2008) | 17(14) | 8.9±2.0 | 108.8±19.1 | NA | 15(12) | 9.7±1.7 | 109.8±19.2 | VBM/gray matter volume | 1.5 T | SPM5 | 8mm | P < 0.001 (uncorrected) | DSM-4 | 9.5 |
| (Kwon, Ow, Pedatella, Lotspeich, & Reiss, 2004) | 11(11) | 13.5±2.3 | NA | NA | 13(13) | 13.6±3.1 | NA | VBM/gray matter volume | 3.0 T | SPM99 | 8mm | P < 0.05 (corrected) | DSM-4 | 9.0 |
| (Mengotti et al., 2011) | 20(18) | 7.0±2.8 | NA | NA | 22(20) | 7.7±2.0 | NA | VBM/gray matter volume | 1.5 T | SPM5 | 8mm | P < 0.001 (uncorrected) | DSM-4 and ADOS | 8.5 |

***Note:*** VBM = voxel-based morphometry; ASD = autism spectrum disorder; TD = typically developing; SD = standard deviation; IQ = intelligence quotient; FWHM = full width at half maximum; NA = not available; SPM = statistical parametric mapping; DPABI = Data Processing & Analysis for Brain Imaging; DPARSF = data processing assistant for resting-State fMRI software; REST = Resting State fMRI Data Analysis Toolkit; ANFI = Analysis of Functional NeuroImages; BAMM = Brain Analysis Morphological Mapping; FSL = FMRIB's Software Library, the University of Oxford; FWE = family wise error; FDR = false discovery rate; GRF = Gaussian random field; TFCE = the Threshold-Free Cluster Enhancement; DSM = Diagnostic and Statistical Manual of Mental Disorders; ICBM = International Consortium for Brain Mapping; ADI-R = Autism Diagnostic Interview-Revised; ADOS = Autism Diagnostic Observation Schedule; ABC = Autism Behavior Checklist; RAADS-R = Ritvo Autism and Asperger Diagnostic Scale Revised; ICD = international Classification of diseases.

**Table S4 Functional subgroup meta-analyses results between ASD and TD**

| **Local Maximum** |  |  |  |  | **Cluster** |  |
| --- | --- | --- | --- | --- | --- | --- |
| **Region** | **Peak MNI coordinate**  **(x, y, z)** | **SDM-Z value** | ***p* value** |  | **No. of voxels** | **Breakdown (No. of voxels)** |
| **Male subgroup**  **ASD < TD** |  |  |  |  |  |  |
| Right inferior temporal gyrus, BA 20 | 48, -4, -38 | -1.846 | <0.001 |  | 604 | Right inferior temporal gyrus, BA 20, 21 (283)  Right temporal pole, middle temporal gyrus, BA 20, 21, 38 (199)  Right temporal pole, superior temporal gyrus, BA 38, 20, 21 (24) |
| Left insula, BA 48 | -40, -10, 11 | -2.145 | <0.001 |  | 361 | Left rolandic operculum, BA 48 (114)  Left insula, BA 48 (29)  Left precentral gyrus, BA 6 (23)  Left superior temporal gyrus, BA 48 (6) |
| Left middle temporal gyrus, BA 21 | -54, -50, 8 | -1.993 | <0.001 |  | 149 | Left middle temporal gyrus, BA 21, 22, 37 (120) |
| Left anterior cingulate / paracingulate gyri, BA 10 | -4, 54, -2 | -1.551 | 0.003 |  | 137 | Right superior frontal gyrus, medial, BA 11, 10 (41)  Left superior frontal gyrus, medial, BA 11, 10 (32)  Left anterior cingulate / paracingulate gyri, BA 10 (22)  Right superior frontal gyrus, medial, BA 10 (7) |
| **ASD > TD** |  |  |  |  |  |  |
| Right precuneus, BA 18 | 10, -96, -4 | 1.552 | <0.001 |  | 861 | Right precuneus, BA 18, 17 (255)  Left precuneus, BA 18, 17 (156) |
| Right angular gyrus, BA 39 | 42, -60, 46 | 1.230 | 0.002 |  | 271 | Right angular gyrus, BA 39, 7, 40 (136) |
| **Childhood subgroup** |  |  |  |  |  |  |
| **ASD < TD** |  |  |  |  |  |  |
| Left insula, BA 48 | -40, -10, 10 | -2.200 | <0.001 |  | 1390 | Left insula, BA 48 (456)  Left rolandic operculum, BA 48 (311)  Left superior temporal gyrus, BA 48 (200) |
| Right superior frontal gyrus, medial, BA 10 | 2, 46, -4 | -1.900 | <0.001 |  | 542 | Right superior frontal gyrus, medial, BA 10, 11 (309)  Left superior frontal gyrus, medial, BA 10, 11 (104)  Left anterior cingulate / paracingulate gyri, BA 10 (71)  Right anterior cingulate / paracingulate gyri, BA 10, 11 (12) |
| Right inferior temporal gyrus, BA 20 | 46, 6, -36 | -1.951 | <0.001 |  | 450 | Right temporal pole, middle temporal gyrus, BA 20, 21, 38 (167)  Right inferior temporal gyrus, BA 20, 21 (144) |
| Left angular gyrus, BA 39 | -44, -56, 28 | -2.280 | <0.001 |  | 321 | Left angular gyrus, BA 39, 40 (188) |
| Right insula, BA 48 | 34, -6, 16 | -1.805 | 0.001 |  | 115 | Right insula, BA 48 (53) |
| Left middle temporal gyrus, BA 21 | -48, -48, 6 | -1.835 | <0.001 |  | 66 | Left middle temporal gyrus, BA 21 (44) |
| **ASD > TD** |  |  |  |  |  |  |
| Right precuneus, BA 17 | 12, -92, 0 | 2.419 | <0.001 |  | 1373 | Right precuneus, BA 17, 18 (334)  Left precuneus, BA 48 (233) |
| Right supplementary motor area, BA 6 | 8, 8, 52 | 1.381 | <0.001 |  | 135 | Right supplementary motor area, BA 6, 32 (86) |
| **Subgroup of ASD without any psychiatric comorbidities** | | | | | | |
| **ASD < TD** |  |  |  |  |  |  |
| Left insula, BA 48 | -36, -10, 12 | -2.205 | <0.001 |  | 1096 | Left insula, BA 48 (414)  Left superior temporal gyrus, BA 48 (59) |
| Right superior frontal gyrus, medial, BA 10 | 2, 50, -2 | -2.050 | <0.001 |  | 948 | Right superior frontal gyrus, medial, BA 10, 11 (474)  Left superior frontal gyrus, medial, BA 11, 10 (213)  Left anterior cingulate / paracingulate gyri, BA 10, 32, 11 (155)  Right anterior cingulate / paracingulate gyri, BA 10, 32, 11 (62) |
| Right middle temporal gyrus, BA 20 | 46, 4, -34 | -1.827 | <0.001 |  | 506 | Right inferior temporal gyrus, BA 20, 21 (236)  Right middle temporal gyrus, BA 20, 21, 38 (196) |
| Left angular gyrus, BA 39 | -44, -56, 28 | -2.013 | <0.001 |  | 213 | Left angular gyrus, BA 39 (145) |
| Right insula, BA 48 | 34, -6, 16 | -1.668 | 0.001 |  | 96 | Right insula, BA 48 (50) |
| **ASD > TD** |  |  |  |  |  |  |
| Right precuneus, BA 18 | 8, -92, 10 | 1.395 | <0.001 |  | 1026 | Left precuneus, BA 18, 17 (302)  Right precuneus, BA 18, 17 (279) |
| Right supplementary motor area, BA 6 | 8, 8, 52 | 1.339 | <0.001 |  | 529 | Right supplementary motor area, BA 6. 8, 32 (234) |
| Left cerebellum, hemispheric lobule VI, BA 18 | -12, -72, -18 | 1.421 | <0.001 |  | 309 | Left cerebellum, hemispheric lobule VI, BA 18, 17, crus I (288) |
| Right fusiform gyrus, BA 19 | 28, -68, -14 | 1.220 | 0.001 |  | 186 | Right fusiform gyrus, BA 19, 18, 37 (135) |
| **Subgroup excluding the insufficient sample size (n < 20) or uncorrected for results** | | | | | | |
| **ASD < TD** |  |  |  |  |  |  |
| Left anterior cingulate / paracingulate gyri, BA 10 | 0, 50, 2 | -1.743 | <0.001 |  | 551 | Right superior frontal gyrus, medial, BA 10 (314)  Left superior frontal gyrus, medial orbital, BA 10 (126)  Left anterior cingulate / paracingulate gyri, BA 10, 32 (80)  Right anterior cingulate / paracingulate gyri, BA 32 (16) |
| Right supplementary motor area, BA 4 | 4, -24, 64 | -1.664 | 0.001 |  | 311 | Right supplementary motor area, BA 4 (76) |
| Left insula, BA 48 | -40, -10, 10 | -1.526 | 0.002 |  | 152 | Left insula, BA 48 (71)  Left rolandic operculum, BA 48 (70)  Left superior temporal gyrus, BA 48 (3) |
| Left angular gyrus, BA 39 | -46, -58, 42 | -1.790 | 0.003 |  | 139 | Left angular gyrus, BA 39, 40 (92) |
| **ASD > TD** |  |  |  |  |  |  |
| Right cerebellum, hemispheric lobule IX | 10, -60, -44 | 1.120 | 0.002 |  | 530 | Right cerebellum, hemispheric lobule IX, VIII (324) |
| Right supplementary motor area, BA 6 | 8, 8, 52 | 1.404 | <0.001 |  | 469 | Right supplementary motor area, BA 6, 8 (237) |
| **ReHo** | | | | | | |
| **ASD < TD** |  |  |  |  |  |  |
| Left insula, BA 48 | -38, -4, 14 | -2.400 | <0.001 |  | 2009 | Left insula, BA 48 (1071)  Left superior temporal gyrus, BA 48 (282) |
| Right superior frontal gyrus, medial, BA 10 | 2, 50, 2 | -1.891 | <0.001 |  | 436 | Right superior frontal gyrus, medial, BA 10, 11 (251)  Left anterior cingulate / paracingulate gyri, BA 10, 32 (76)  Left superior frontal gyrus, medial, BA 10, 11 (84) |
| Right insula, BA 48 | 36, -4, 12 | -1.722 | 0.002 |  | 207 | Right insula, BA 48 (112) |
| Left angular gyrus, BA 39 | -44, -56, 30 | -2.125 | <0.001 |  | 150 | Left angular gyrus, BA 39 (89) |
| **ASD > TD** |  |  |  |  |  |  |
| Left precuneus, BA 17 | -2, -88, 8 | 1.564 | <0.001 |  | 499 | Left precuneus, BA 17, 18 (265)  Right precuneus, BA 17, 18 (132) |
| Right fusiform gyrus, BA 19 | 26, -68, -12 | 1.604 | <0.001 |  | 312 | Right fusiform gyrus, BA 19, 18, 37 (167)  Right cerebellum, hemispheric lobule VI, BA 19, 18 (81)  Right lingual gyrus, BA 18 (29) |
| Right supplementary motor area, BA 8 | 10, 22, 56 | 1.509 | <0.001 |  | 196 | Right supplementary motor area, BA 8, 6 (59) |

***Note:*** ASD = autism spectrum disorder; TD = typically developing; MNI = Montreal Neurological Institute; SDM = signed differential mapping; BA = Brodmann area; ReHo = regional homogeneity.

**Table S5 VBM subgroup meta-analyses results between ASD and TD**

| **Local Maximum** |  |  |  |  | **Cluster** |  |
| --- | --- | --- | --- | --- | --- | --- |
| **Region** | **Peak MNI coordinate**  **(x, y, z)** | **SDM-Z value** | ***p* value** |  | **No. of voxels** | **Breakdown (No. of voxels)** |
| **Male subgroup** |  |  |  |  |  |  |
| **ASD < TD** |  |  |  |  |  |  |
| Left inferior parietal gyri, BA 7 | -28, -74, 46 | -1.511 | <0.001 |  | 253 | Left inferior parietal gyri, BA 7, 19 (94) |
| **ASD > TD** |  |  |  |  |  |  |
| Left insula, BA 48 | -42, -16, 2 | 2.434 | <0.001 |  | 876 | Left insula, BA 48 (291)  Left rolandic operculum, BA 48 (123)  Left superior temporal gyrus, BA 48 (115) |
| Left middle temporal gyrus, BA 21 | -44, 6, 18 | 2.274 | <0.001 |  | 302 | Left middle temporal gyrus, BA 21, 20 (99)  Left temporal pole, superior temporal gyrus, BA 38, 21, 48, 20 (91) |
| Left olfactory cortex, BA 25 | -6, 20, -8 | 1.767 | 0.001 |  | 223 | Left olfactory cortex, BA 25 (66)  Right olfactory cortex, BA 25 (27)  Left striatum (31) |
| **Childhood subgroup** |  |  |  |  |  |  |
| **ASD < TD** |  |  |  |  |  |  |
| Left cerebellum, hemispheric lobule VIII | -8, -66, -44 | -1.181 | <0.001 |  | 1843 | Left cerebellum, hemispheric lobule VIII, IX, VIIB, crus II, crus I (1044)  Cerebellum, vermic lobule VIII, IX (228)  Right cerebellum, hemispheric lobule IX, VIII (112) |
| Right insula, BA 48 | 36, -18, 8 | -1.063 | 0.001 |  | 182 | Right insula, BA 48 (103) |
| **ASD > TD** |  |  |  |  |  |  |
| Left insula, BA 48 | -42, -16, 2 | 1.659 | <0.001 |  | 264 | Left superior temporal gyrus, BA 48 (66)  Left insula, BA 48 (44) |
| Left cuneus cortex, BA 18 | -10, -90, 22 | 1.544 | <0.001 |  | 203 | Left cuneus cortex, BA 18, 19 (91)  Right cuneus cortex, BA 18 (9) |
| Right angular gyrus, BA 7 | 40, -68, 36 | 1.366 | 0.002 |  | 106 | Right angular gyrus, BA 7, 39, 19 (70) |
| **Adolescence subgroup** |  |  |  |  |  |  |
| **ASD < TD** |  |  |  |  |  |  |
| Left anterior cingulate / paracingulate gyri | 0, 0, 30 | -1.678 | <0.001 |  | 534 | Left median cingulate / paracingulate gyri, BA 24, 23 (199)  Right median cingulate / paracingulate gyri, BA 24, 23, 32 (192)  Left anterior cingulate / paracingulate gyri, BA 24 (23)  Right anterior cingulate / paracingulate gyri, BA 24 (1) |
| **ASD > TD** |  |  |  |  |  |  |
| Cerebellum, vermic lobule IX | 2, -60, -42 | 2.138 | <0.001 |  | 906 | Cerebellum, vermic lobule VIII, IX, VII (337)  Right cerebellum, hemispheric lobule VIII, IX, VI, VIIB, crus II (233)  Left cerebellum, hemispheric lobule VIII, IX, crus II (146) |
| Left olfactory cortex, BA 25 | 0, 16, -8 | 2.361 | <0.001 |  | 590 | Left olfactory cortex, BA 25, 11 (167)  Right olfactory cortex, BA 25, 11 (66) |
| **Adult ASD subgroup** | | | | | | |
| **ASD < TD** |  |  |  |  |  |  |
| Left anterior cingulate / paracingulate gyri, BA 32 | 0, 40, 14 | -2.143 | <0.001 |  | 1226 | Left anterior cingulate / paracingulate gyri, BA 32, 24, 10, 25 (516)  Right anterior cingulate / paracingulate gyri, BA 32, 24, 10, 25 (345)  Left superior frontal gyrus, medial, BA 32, 10, 9 (220)  Right superior frontal gyrus, medial BA 10, 32 (37) |
| Right inferior temporal gyrus, BA 37 | 54, -58, -12 | -1.740 | <0.001 |  | 295 | Right inferior temporal gyrus, BA 37, 20, 19, 21 (273) |
| Right precuneus, BA 17 | 6, -72, 12 | -1.505 | 0.001 |  | 146 | Left precuneus, BA 17, 18, 23 (77)  Right precuneus, BA 17, 18 (46) |
| **ASD > TD** |  |  |  |  |  |  |
| Left middle temporal gyrus, BA 21 | -62, -22, -14 | 2.319 | <0.001 |  | 1433 | Left middle temporal gyrus, BA 21, 20, 22, 48 (863)  Left superior temporal gyrus, BA 48, 22 (81)  Left inferior temporal gyrus, BA 20, 21 (74) |
| Right precentral gyrus, BA 6 | 52, -2, 40 | 1.546 | <0.001 |  | 745 | Right precentral gyrus, BA 6, 4, 3 (482)  Right postcentral gyrus, BA 4, 3, 6 (222) |
| Left precentral gyrus, BA 6 | -38, -16, 50 | 1.603 | <0.001 |  | 462 | Left precentral gyrus, BA 6, 4, 3 (210)  Left postcentral gyrus, BA 4, 6 (209) |
| **Subgroup of ASD without any psychiatric comorbidities** | | | | | | |
| **ASD < TD** |  |  |  |  |  |  |
| Left cerebellum, hemispheric lobule VIIB | -40, -56, -46 | -1.484 | <0.001 |  | 841 | Left cerebellum, crus II, I, hemispheric lobule VIIB, VIII (825) |
| Left anterior cingulate / paracingulate gyri, BA 32 | 0, 44, 18 | -1.300 | 0.002 |  | 105 | Left anterior cingulate / paracingulate gyri, BA 32, 24 (86)  Left superior frontal gyrus, medial, BA 32 (5) |
| **ASD > TD** |  |  |  |  |  |  |
| Left middle temporal gyrus, BA 21 | -60, -20, -14 | 2.106 | <0.001 |  | 1060 | Left middle temporal gyrus, BA 21, 20, 22, 48 (380) |
| Left olfactory cortex, BA 25 | -2, 20, -8 | 1.797 | <0.001 |  | 238 | Left olfactory cortex, BA 25, 11 (87)  Right olfactory cortex, BA 25 (9) |
| Right precentral gyrus, BA 6 | 42, 2, 38 | 1.826 | <0.001 |  | 126 | Right precentral gyrus, BA 6, 44 (46) |
| Left middle frontal gyrus, BA 9 | -20, 44, 32 | 1.763 | <0.001 |  | 89 | Left middle frontal gyrus, BA 9, 46 (39)  Left superior frontal gyrus, BA 9 (27) |
| **Subgroup excluding the insufficient sample size (n < 20) or uncorrected for results** | | | | | | |
| **ASD < TD** |  |  |  |  |  |  |
| Left anterior cingulate / paracingulate gyri | 0, 0, 30 | -1.968 | <0.001 |  | 587 | Left median cingulate / paracingulate gyri, BA 24, 23, 32 (287)  Right median cingulate / paracingulate gyri, BA 24 (150)  Left anterior cingulate / paracingulate gyri, BA 24 (70)  Right anterior cingulate / paracingulate gyri, BA 24 (15) |
| Left cerebellum, crus I |  | -1.302 | 0.002 |  | 610 | Left cerebellum, crus I, II (582) |
| Left superior parietal gyrus, BA 7 | -30, -66, 48 | -1.334 | 0.002 |  | 288 | Left superior parietal gyrus, BA 7 (130) |
| **ASD > TD** |  |  |  |  |  |  |
| Left olfactory cortex, BA 25 | 2, 16, -4 | 1.936 | <0.001 |  | 616 | Left olfactory cortex, BA 25, 11 (106)  Right olfactory cortex, BA 25, 11 (63) |
| Cerebellum, vermic lobule VIII | -2, -60, -34 | 1.466 | <0.001 |  | 538 | Cerebellum, vermic lobule VIII, IX (261)  Right cerebellum, hemispheric lobule VIII, IX (87)  Left cerebellum, hemispheric lobule VIII, IX (58) |
| Right cerebellum, hemispheric lobule III | 12, -32, -22 | 1.209 | 0.003 |  | 60 | Right cerebellum, hemispheric lobule III, BA 30 (17) |
| Left middle temporal gyrus, BA 39 | -56, -60, 22 | 1.215 | 0.003 |  | 59 | Left middle temporal gyrus, BA 21, 39, 22, 37 (27) |

***Note:*** VBM = voxel-based morphometry; ASD = autism spectrum disorder; TD = typically developing; MNI = Montreal Neurological Institute; SDM = signed differential mapping; BA = Brodmann area.

**Table S6 Meta-regression analyses: factors affecting resting-state functional activity in studies of** individuals **with ASD**

|  | **Region** | **Peak MNI coordinate**  **(x, y, z)** | **No. of voxels** | **SDM-Z value** | ***p* value** |
| --- | --- | --- | --- | --- | --- |
| **Total** individuals **with ASD** | | | | | |
| Effects of ADI-R score | Right inferior temporal gyrus, BA 21, 20 | 48, 10, -28 | 96 | -2.932 | <0.001 |

***Note:*** ASD = autism spectrum disorder; MNI = Montreal Neurological Institute; SDM = signed differential mapping; ADI-R = Autism Diagnostic Interview—Revised; BA = Brodmann area.

**Table S7 Meta-regression analyses: factors affecting GMV in studies of** individuals **with ASD**

|  | **Region** | **Peak MNI coordinate**  **(x, y, z)** | **No. of voxels** | **SDM-Z value** | ***p* value** |
| --- | --- | --- | --- | --- | --- |
| **Total** individuals **with ASD** | | | | | |
| Effects of age | Left middle temporal gyrus, BA 21 | -62, -22, -14 | 325 | 1.848 | <0.001 |
|  | Left anterior cingulate / paracingulate gyri, BA 32 | 0, 42, 14 | 106 | -1.821 | <0.001 |

***Note:*** GMV = gray matter volume; ASD = autism spectrum disorder; MNI = Montreal Neurological Institute; SDM = signed differential mapping; ADI-R = Autism Diagnostic Interview—Revised; BA = Brodmann area.

**Supplementary references**

Abell, F., Krams, M., Ashburner, J., Passingham, R., Friston, K., Frackowiak, R., . . . & Frith, U. (1999). The neuroanatomy of autism: A voxel-based whole brain analysis of structural scans. *Neuroreport, 10*(8), 1647-1651

Albajara Saenz, A., Van Schuerbeek, P., Baijot, S., Septier, M., Deconinck, N., Defresne, P., . . . & Massat, I. (2020). Disorder-specific brain volumetric abnormalities in attention-deficit/hyperactivity disorder relative to autism spectrum disorder. *PLoS One, 15*(11), e0241856

Albajes-Eizagirre, A., Solanes, A., Vieta, E., & Radua, J. (2019). Voxel-based meta-analysis via permutation of subject images (psi): Theory and implementation for sdm. *Neuroimage, 186*, 174-184

Anteraper, S. A., Guell, X., Hollinshead, M. O., D'Mello, A., Whitfield-Gabrieli, S., Biederman, J., & Joshi, G. (2020). Functional alterations associated with structural abnormalities in adults with high-functioning autism spectrum disorder. *Brain Connect, 10*(7), 368-376

Bonilha, L., Cendes, F., Rorden, C., Eckert, M., Dalgalarrondo, P., Li, L. M., & Steiner, C. E. (2008). Gray and white matter imbalance--typical structural abnormality underlying classic autism? *Brain Dev, 30*(6), 396-401

Brieber, S., Neufang, S., Bruning, N., Kamp-Becker, I., Remschmidt, H., Herpertz-Dahlmann, B., . . . & Konrad, K. (2007). Structural brain abnormalities in adolescents with autism spectrum disorder and patients with attention deficit/hyperactivity disorder. *J Child Psychol Psychiatry, 48*(12), 1251-1258

Cai, J., Hu, X., Guo, K., Yang, P., Situ, M., & Huang, Y. (2018). Increased left inferior temporal gyrus was found in both low function autism and high function autism. *Front Psychiatry, 9*, 542

Cao, X.H, Zhang, AX, Liu, ZF, & Yang, CX. (2016). A resting-state fmri study in adolescents with autism spectrum disorder. *Chin J Clin, 10(3):325-330*

Cheng H, Gong GL, Peng Y, Cao XH, Zhang AX, Liu ZF, . . . & Liu ZF. (2016). Abnormalities of spontaneous neuronal activity low-frequency fluctuation of typical autistic children: A functional magnetic resonance imaging study. *Chin Mental Health J, 30*(3), 441-447

Cheng, Y., Chou, K. H., Fan, Y. T., & Lin, C. P. (2011). Ans: Aberrant neurodevelopment of the social cognition network in adolescents with autism spectrum disorders. *PLoS One, 6*(4), e18905

Craig, M. C., Zaman, S. H., Daly, E. M., Cutter, W. J., Robertson, D. M., Hallahan, B., . . . & Murphy, D. G. (2007). Women with autistic-spectrum disorder: Magnetic resonance imaging study of brain anatomy. *Br J Psychiatry, 191*, 224-228

David, N., Schultz, J., Milne, E., Schunke, O., Schottle, D., Munchau, A., . . . & Engel, A. K. (2014). Right temporoparietal gray matter predicts accuracy of social perception in the autism spectrum. *J Autism Dev Disord, 44*(6), 1433-1446

Du L. (2021). Multimodal magnetic resonance imaging in autsim spectrum disorders and alzheimer's disease. *Doctor thesis Beijing: Peking Union Medical College*

Ecker, C., Rocha-Rego, V., Johnston, P., Mourao-Miranda, J., Marquand, A., Daly, E. M., . . . & Consortium, Mrc Aims. (2010). Investigating the predictive value of whole-brain structural mr scans in autism: A pattern classification approach. *Neuroimage, 49*(1), 44-56

Ecker, C., Suckling, J., Deoni, S. C., Lombardo, M. V., Bullmore, E. T., Baron-Cohen, S., . . . & Consortium, Mrc Aims. (2012). Brain anatomy and its relationship to behavior in adults with autism spectrum disorder: A multicenter magnetic resonance imaging study. *Arch Gen Psychiatry, 69*(2), 195-209

Foster, N. E., Doyle-Thomas, K. A., Tryfon, A., Ouimet, T., Anagnostou, E., Evans, A. C., . . . & NeuroDevNet, A. S. D. imaging group. (2015). Structural gray matter differences during childhood development in autism spectrum disorder: A multimetric approach. *Pediatr Neurol, 53*(4), 350-359

Freitag, C. M., Konrad, C., Haberlen, M., Kleser, C., von Gontard, A., Reith, W., . . . & Krick, C. (2008). Perception of biological motion in autism spectrum disorders. *Neuropsychologia, 46*(5), 1480-1494

Greimel, E., Nehrkorn, B., Schulte-Ruther, M., Fink, G. R., Nickl-Jockschat, T., Herpertz-Dahlmann, B., . . . & Eickhoff, S. B. (2013). Changes in grey matter development in autism spectrum disorder. *Brain Struct Funct, 218*(4), 929-942

Guo, X., Duan, X., Suckling, J., Wang, J., Kang, X., Chen, H., . . . & Chen, H. (2021). Mapping progressive gray matter alterations in early childhood autistic brain. *Cereb Cortex, 31*(3), 1500-1510

Hao GF. (2020). Resting-state functional magnetic resonance imaging study of scalp acupuncture in the treatment of children with autism spectrum disorders. *Doctor thesis. Shan Dong: SHANDONG UNIVERSITY*

Hyde, K. L., Samson, F., Evans, A. C., & Mottron, L. (2010). Neuroanatomical differences in brain areas implicated in perceptual and other core features of autism revealed by cortical thickness analysis and voxel-based morphometry. *Hum Brain Mapp, 31*(4), 556-566

Itahashi, T., Yamada, T., Watanabe, H., Nakamura, M., Ohta, H., Kanai, C., . . . & Hashimoto, R. (2015). Alterations of local spontaneous brain activity and connectivity in adults with high-functioning autism spectrum disorder. *Mol Autism, 6*, 30

Jann, K., Hernandez, L. M., Beck-Pancer, D., McCarron, R., Smith, R. X., Dapretto, M., & Wang, D. J. (2015). Altered resting perfusion and functional connectivity of default mode network in youth with autism spectrum disorder. *Brain Behav, 5*(9), e00358

Jao Keehn, R. J., Nair, S., Pueschel, E. B., Linke, A. C., Fishman, I., & Muller, R. A. (2019). Atypical local and distal patterns of occipito-frontal functional connectivity are related to symptom severity in autism. *Cereb Cortex, 29*(8), 3319-3330

Katz, J., d'Albis, M. A., Boisgontier, J., Poupon, C., Mangin, J. F., Guevara, P., . . . & Houenou, J. (2016). Similar white matter but opposite grey matter changes in schizophrenia and high-functioning autism. *Acta Psychiatr Scand, 134*(1), 31-39

Kaufmann, L., Zotter, S., Pixner, S., Starke, M., Haberlandt, E., Steinmayr-Gensluckner, M., . . . & Marksteiner, J. (2013). Brief report: Cantab performance and brain structure in pediatric patients with asperger syndrome. *J Autism Dev Disord, 43*(6), 1483-1490

Ke, X., Hong, S., Tang, T., Zou, B., Li, H., Hang, Y., . . . & Liu, Y. (2008). Voxel-based morphometry study on brain structure in children with high-functioning autism. *Neuroreport, 19*(9), 921-925

Kojima, M., Yassin, W., Owada, K., Aoki, Y., Kuwabara, H., Natsubori, T., . . . & Yamasue, H. (2019). Neuroanatomical correlates of advanced paternal and maternal age at birth in autism spectrum disorder. *Cereb Cortex, 29*(6), 2524-2532

Kosaka, H., Omori, M., Munesue, T., Ishitobi, M., Matsumura, Y., Takahashi, T., . . . & Wada, Y. (2010). Smaller insula and inferior frontal volumes in young adults with pervasive developmental disorders. *Neuroimage, 50*(4), 1357-1363

Kurth, F., Narr, K. L., Woods, R. P., O'Neill, J., Alger, J. R., Caplan, R., . . . & Levitt, J. G. (2011). Diminished gray matter within the hypothalamus in autism disorder: A potential link to hormonal effects? *Biol Psychiatry, 70*(3), 278-282

Kwon, H., Ow, A. W., Pedatella, K. E., Lotspeich, L. J., & Reiss, A. L. (2004). Voxel-based morphometry elucidates structural neuroanatomy of high-functioning autism and asperger syndrome. *Dev Med Child Neurol, 46*(11), 760-764

Lai, M. C., Lombardo, M. V., Suckling, J., Ruigrok, A. N., Chakrabarti, B., Ecker, C., . . . & Baron-Cohen, S. (2013). Biological sex affects the neurobiology of autism. *Brain, 136*(Pt 9), 2799-2815

Lan, Z., Xu, S., Wu, Y., Xia, L., Hua, K., Li, M., . . . & Wang, T. (2021). Alterations of regional homogeneity in preschool boys with autism spectrum disorders. *Front Neurosci, 15*, 644543

Li, G., Rossbach, K., Jiang, W., & Du, Y. (2018). Resting-state brain activity in chinese boys with low functioning autism spectrum disorder. *Ann Gen Psychiatry, 17*, 47

Li, G., Rossbach, K., Jiang, W., Zhao, L., Zhang, K., & Du, Y. (2019). Reduction in grey matter volume and its correlation with clinical symptoms in chinese boys with low functioning autism spectrum disorder. *J Intellect Disabil Res, 63*(2), 113-123

Li X, Liu J, Yang W, Li X, Liu J, Yang W, . . . & Yang W. (2013). A resting-state functional magnetic resonance imaging study of brain activity in children with autism. *Chin J Psychiatry, 46*, 137-141

Li Y, Zhang L, Xu H, & Jiang Q. (2022). Differences of brain function between autism spectrum disorder children with and without depression during rest. *Chin J Nerv Ment Dis, 48*, 84-89

Lim, L., Chantiluke, K., Cubillo, A. I., Smith, A. B., Simmons, A., Mehta, M. A., & Rubia, K. (2015). Disorder-specific grey matter deficits in attention deficit hyperactivity disorder relative to autism spectrum disorder. *Psychol Med, 45*(5), 965-976

Lin, H. Y., Ni, H. C., Lai, M. C., Tseng, W. I., & Gau, S. S. (2015). Regional brain volume differences between males with and without autism spectrum disorder are highly age-dependent. *Mol Autism, 6*, 29

Lin, H. Y., Tseng, W. I., Lai, M. C., Chang, Y. T., & Gau, S. S. (2017). Shared atypical brain anatomy and intrinsic functional architecture in male youth with autism spectrum disorder and their unaffected brothers. *Psychol Med, 47*(4), 639-654

Maximo, J. O., Keown, C. L., Nair, A., & Muller, R. A. (2013). Approaches to local connectivity in autism using resting state functional connectivity mri. *Front Hum Neurosci, 7*, 605

McAlonan, G. M., Daly, E., Kumari, V., Critchley, H. D., van Amelsvoort, T., Suckling, J., . . . & Murphy, D. G. (2002). Brain anatomy and sensorimotor gating in asperger's syndrome. *Brain, 125*(Pt 7), 1594-1606

McAlonan, G. M., Suckling, J., Wong, N., Cheung, V., Lienenkaemper, N., Cheung, C., & Chua, S. E. (2008). Distinct patterns of grey matter abnormality in high-functioning autism and asperger's syndrome. *J Child Psychol Psychiatry, 49*(12), 1287-1295

Mei, T., Ma, Z. H., Guo, Y. Q., Lu, B., Cao, Q. J., Chen, X., . . . & Liu, J. (2022). Frequency-specific age-related changes in the amplitude of spontaneous fluctuations in autism. *Transl Pediatr, 11*(3), 349-358

Mengotti, P., D'Agostini, S., Terlevic, R., De Colle, C., Biasizzo, E., Londero, D., . . . & Brambilla, P. (2011). Altered white matter integrity and development in children with autism: A combined voxel-based morphometry and diffusion imaging study. *Brain Res Bull, 84*(2), 189-195

Mueller, S., Keeser, D., Samson, A. C., Kirsch, V., Blautzik, J., Grothe, M., . . . & Meindl, T. (2013). Convergent findings of altered functional and structural brain connectivity in individuals with high functioning autism: A multimodal mri study. *PLoS One, 8*(6), e67329

Ni, H. C., Lin, H. Y., Tseng, W. I., Chiu, Y. N., Wu, Y. Y., Tsai, W. C., & Gau, S. S. (2018). Neural correlates of impaired self-regulation in male youths with autism spectrum disorder: A voxel-based morphometry study. *Prog Neuropsychopharmacol Biol Psychiatry, 82*, 233-241

Noppari, T., Sun, L., Lukkarinen, L., Putkinen, V., Tani, P., Lindberg, N., . . . & Nummenmaa, L. (2022). Brain structural alterations in autism and criminal psychopathy. *Neuroimage Clin, 35*, 103116

Paakki, J. J., Rahko, J., Long, X., Moilanen, I., Tervonen, O., Nikkinen, J., . . . & Kiviniemi, V. (2010). Alterations in regional homogeneity of resting-state brain activity in autism spectrum disorders. *Brain Res, 1321*, 169-179

Pagani, M., Manouilenko, I., Stone-Elander, S., Odh, R., Salmaso, D., Hatherly, R., . . . & Bejerot, S. (2012). Brief report: Alterations in cerebral blood flow as assessed by pet/ct in adults with autism spectrum disorder with normal iq. *J Autism Dev Disord, 42*(2), 313-318

Poulin-Lord, M. P., Barbeau, E. B., Soulieres, I., Monchi, O., Doyon, J., Benali, H., & Mottron, L. (2014). Increased topographical variability of task-related activation in perceptive and motor associative regions in adult autistics. *Neuroimage Clin, 4*, 444-453

Poustka, L., Jennen-Steinmetz, C., Henze, R., Vomstein, K., Haffner, J., & Sieltjes, B. (2012). Fronto-temporal disconnectivity and symptom severity in children with autism spectrum disorder. *World J Biol Psychiatry, 13*(4), 269-280

Qiu XY. (2009). Research on brain function of childhood autism. *Master thesis. Guang Dong: Sun Yat-sen University*

Radeloff, D., Ciaramidaro, A., Siniatchkin, M., Hainz, D., Schlitt, S., Weber, B., . . . & Freitag, C. M. (2014). Structural alterations of the social brain: A comparison between schizophrenia and autism. *PLoS One, 9*(9), e106539

Radua, J., Mataix-Cols, D., Phillips, M. L., El-Hage, W., Kronhaus, D. M., Cardoner, N., & Surguladze, S. (2012). A new meta-analytic method for neuroimaging studies that combines reported peak coordinates and statistical parametric maps. *Eur Psychiatry, 27*(8), 605-611

Retico, A., Giuliano, A., Tancredi, R., Cosenza, A., Apicella, F., Narzisi, A., . . . & Calderoni, S. (2016). The effect of gender on the neuroanatomy of children with autism spectrum disorders: A support vector machine case-control study. *Mol Autism, 7*, 5

Riedel, A., Maier, S., Ulbrich, M., Biscaldi, M., Ebert, D., Fangmeier, T., . . . & Tebartz van Elst, L. (2014). No significant brain volume decreases or increases in adults with high-functioning autism spectrum disorder and above average intelligence: A voxel-based morphometric study. *Psychiatry Res, 223*(2), 67-74

Riva, D., Annunziata, S., Contarino, V., Erbetta, A., Aquino, D., & Bulgheroni, S. (2013). Gray matter reduction in the vermis and crus-ii is associated with social and interaction deficits in low-functioning children with autistic spectrum disorders: A vbm-dartel study. *Cerebellum, 12*(5), 676-685

Rojas, D. C., Peterson, E., Winterrowd, E., Reite, M. L., Rogers, S. J., & Tregellas, J. R. (2006). Regional gray matter volumetric changes in autism associated with social and repetitive behavior symptoms. *BMC Psychiatry, 6*, 56

Sato, W., Kochiyama, T., Uono, S., Yoshimura, S., Kubota, Y., Sawada, R., . . . & Toichi, M. (2017). Reduced gray matter volume in the social brain network in adults with autism spectrum disorder. *Front Hum Neurosci, 11*, 395

Schmitz, N., Rubia, K., Daly, E., Smith, A., Williams, S., & Murphy, D. G. (2006). Neural correlates of executive function in autistic spectrum disorders. *Biol Psychiatry, 59*(1), 7-16

Seng, G. J., Lai, M. C., Goh, J. O. S., Tseng, W. I., & Gau, S. S. (2022). Gray matter volume alteration is associated with insistence on sameness and cognitive flexibility in autistic youth. *Autism Res, 15*(7), 1209-1221

Toal, F., Bloemen, O. J., Deeley, Q., Tunstall, N., Daly, E. M., Page, L., . . . & Murphy, D. G. (2009). Psychosis and autism: Magnetic resonance imaging study of brain anatomy. *Br J Psychiatry, 194*(5), 418-425

Toal, F., Daly, E. M., Page, L., Deeley, Q., Hallahan, B., Bloemen, O., . . . & Murphy, D. G. (2010). Clinical and anatomical heterogeneity in autistic spectrum disorder: A structural mri study. *Psychol Med, 40*(7), 1171-1181

Waiter, G. D., Williams, J. H., Murray, A. D., Gilchrist, A., Perrett, D. I., & Whiten, A. (2004). A voxel-based investigation of brain structure in male adolescents with autistic spectrum disorder. *Neuroimage, 22*(2), 619-625

Wang, H., Ma, Z. H., Xu, L. Z., Yang, L., Ji, Z. Z., Tang, X. Z., . . . & Liu, J. (2022). Developmental brain structural atypicalities in autism: A voxel-based morphometry analysis. *Child Adolesc Psychiatry Ment Health, 16*(1), 7

Wang, J., Fu, K., Chen, L., Duan, X., Guo, X., Chen, H., . . . & Chen, H. (2017). Increased gray matter volume and resting-state functional connectivity in somatosensory cortex and their relationship with autistic symptoms in young boys with autism spectrum disorder. *Front Physiol, 8*, 588

Wang LL. (2016). A study on regional homogeneity of resting-state brain activity in preschool children with autism spectrum disorder. *Master thesis. Chong Qing: Chongqing Medical University*

Wang, W., Liu, J., Shi, S., Liu, T., Ma, L., Ma, X., . . . & Wang, M. (2018). Altered resting-state functional activity in patients with autism spectrum disorder: A quantitative meta-analysis. *Front Neurol, 9*, 556

Wang Y. (2018). A study on fairness and resting state regional homogeneity of children and adolescents with high-functioning autism spectrum disorder. *Master thesis. Nan Jing: Nanjing Medical University*

Wilson, L. B., Tregellas, J. R., Hagerman, R. J., Rogers, S. J., & Rojas, D. C. (2009). A voxel-based morphometry comparison of regional gray matter between fragile x syndrome and autism. *Psychiatry Res, 174*(2), 138-145

Xiu LJ. (2010). Regional cerebral blood flow in children with autism spectrum disorder a spect study. *Master thesis. Guang Dong: Sun Yat-sen University*

Yang, Q., Huang, P., Li, C., Fang, P., Zhao, N., Nan, J., . . . & Cui, L. B. (2018). Mapping alterations of gray matter volume and white matter integrity in children with autism spectrum disorder: Evidence from fmri findings. *Neuroreport, 29*(14), 1188-1192

Yerys, B. E., Herrington, J. D., Bartley, G. K., Liu, H. S., Detre, J. A., & Schultz, R. T. (2018). Arterial spin labeling provides a reliable neurobiological marker of autism spectrum disorder. *J Neurodev Disord, 10*(1), 32

Yin TN, Li X, Wang H, Cao XH, Zhang AX, Liu ZF, . . . & Liu ZF. (2021). Relationship between theory of mind and local brain functional connectivity children with autism spectrum disorder. *Chinese Mental Health Journal, 35*(3), 954-959

Yue, X., Zhang, G., Li, X., Shen, Y., Wei, W., Bai, Y., . . . & Wang, M. (2022). Brain functional alterations in prepubertal boys with autism spectrum disorders. *Front Hum Neurosci, 16*, 891965

Zhao, X., Zhu, S., Cao, Y., Cheng, P., Lin, Y., Sun, Z., . . . & Du, Y. (2022). Abnormalities of gray matter volume and its correlation with clinical symptoms in adolescents with high-functioning autism spectrum disorder. *Neuropsychiatr Dis Treat, 18*, 717-730

Zhao, X., Zhu, S., Cao, Y., Cheng, P., Lin, Y., Sun, Z., . . . & Du, Y. (2022). Regional homogeneity of adolescents with high-functioning autism spectrum disorder and its association with symptom severity. *Brain Behav, 12*(8), e2693
